# POSTERS

**DOI:** 10.3402/mehd.v23i0.17461

**Published:** 2012-05-23

To overcome shortages of human donor organs for allotransplantation for many organ failure patients, we made commitment to develop gnotobiotic miniature swine for alternative organ donor source for xenotransplantation program.

For this, we have constructed absolute barrier-sustained gnotobiotic facility at Konkuk University, Bio-Organ Research Center, Seoul, Korea, and applied germfree technology. Gnotobiotic miniature swine was originally developed by Dr. Yoon B. Kim of Rosalind Franklin University of Medicine and Science, Chicago Medical School, North Chicago, IL, and donated to Seoul National University College of Medicine (SNU), Seoul, Korea. Pregnant SNU sows were procured from SNU, and germfree piglets were obtained by aseptic hysterectomy. These piglets were maintained in germfree isolators for about 4 weeks, were deprived of colostrums and were fed sterilized soybean milk by gamma-irradiation and they were associated with anaerobic di-flora, *Lactobacillus* sp. and *Streptococcus* sp., confirmed successful associations by rectal swab cultures and transferred into gnotobiotic facility aseptically. They are maintained on Hepa filtered air in and out, maintaining constant pen room air pressure of 0.9±0.1 in., sterile water, and sterilized diet. In 10 sessions of hysterectomy, 18 male and 33 female piglets were obtained of which 8 male and 8 female piglets died within 5 days after birth. Among live piglets, 6 male and 15 female piglets were confirmed to be germfree by microbial monitoring before the association of di-flora. Genotyping for swine leukocyte antigen (SLA) showed 2 males and 7 females to be homozygous for MHC class II (DRB, DQB), 0301, and 1 male and 1 female were homozygous for MHC class II (DRB, DQB), 0201. Molecular genetic and immunological research projects for further development of genetic modifications for humanized and inbred miniature swine for ideal organ donor source for xenotransplantation program are progressing.
