# Supplementary material for: POSTERS
Source: Microb Ecol Health Dis. 2012 May 23;23:10.3402/mehd.v23i0.17461. doi: 10.3402/mehd.v23i0.17461 (PMC3747765; doi:10.3402/mehd.v23i0.17461)
Supplement: Related texts

# Sub article: *Lactobacillus* monoassociated mice might be downregulated by the serum antibody levels induced by dietary antigen compared with *Bacteroides* monoassociated mice

**Published:** 2012-05-23

**Authors:** Yuji Hamamoto, Akira Hosono, Masato Tsuda, Daiki Kamoi, Satoshi Hachimura, Yoshika Momose, Kikuji Itoh, Kazuhiro Hirayama, Kyoko Takahashi, Shuichi Kaminogawa

**Affiliations:** 1 Food and Physiological Functions Laboratory, Department of Food Bioscience and Biotechnology, Nihon University, Kanagawa, Japan; 2 Research Center for Food Safety, Graduate School of Agricultural and Life Science, The University of Tokyo, Tokyo, Japan; 3 Department of Veterinary Public Health, Graduate School of Agricultural and Life Science, The University of Tokyo, Tokyo, Japan

It is thought that colonization of the gut by commensal bacteria modulates the induction of oral tolerance and food allergy. However, it is not known which genera of intestinal commensal bacteria modulate food allergy induced by oral antigens. Therefore, we examined whether the distinct intestinal commensal bacteria have different immunomodulatory effects for food antigen-specific immune responses. In order to investigate this theme, we used ovalbumin-specific T cell receptor transgenic (OVA23-3) mice under the condition that controlled bacterial environment.

We generated monoassociated mice by inoculating germfree (GF) mice of OVA23-3 with either *Lactobacillus johnsonii* 129 (LJ) or *Bacteroides acidifaciens* type A43 (BA) isolated from murine intestine. For monoassociation with BA or LJ, these mice were orally inoculated with approximately 1×10^9^ CFU of BA or LJ at 4–5 weeks of age. Thereafter, BA, LJ and conventional (CV) mice were administered a 20% OVA-containing diet or a casein-containing diet for 4 weeks. Serum antibody titers were measured by ELISA.

There was a tendency for total IgE and OVA-specific IgE levels to be higher in the serum of BA mice fed with the OVA diet than those of CV mice fed the same diet. The OVA-specific IgG2a levels in the serum of BA mice were significantly higher than that of CV mice. However, LJ mice showed similar levels of each serum anti body titers compared with CV mice. These data suggest that distinct intestinal commensal bacteria have different immunomodulatory effects, and *Lactobacillus* monoassociated mice might be downregulated by the serum antibody levels induced by dietary antigen compared with *Bacteroides* monoassociated mice.

# Sub article: High incidence of radiation-induced cavernous hemangioma in long-term survivors who underwent hematopoietic stem cell transplantation with radiation therapy during childhood or adolescence

**Published:** 2012-05-23

**Authors:** Takashi Koike, Noriharu Yanagimachi, Hiroyuki Ishiguro, Hiromasa Yabe, Miharu Yabe, Tsuyoshi Morimoto, Takashi Shimizu, Hiromitsu Takakura, Shunichi Kato

**Affiliations:** 1 Department of Pediatrics, Tokai University School of Medicine, Isehara, Japan; 2 Department of Radiology, Tokai University School of Medicine, Isehara, Japan; 3 Department of Cell Transplantation & Regenerative Medicine, Tokai University School of Medicine, Isehara, Japan; 4 Department of Laboratory Medicine, Tokai University School of Medicine, Isehara, Japan

Radiation-induced cavernous hemangioma (RICH) is a late complication of cerebral radiation therapy. Long-term surviving hematopoietic stem cell transplant (HSCT) recipients who have received radiation therapy could be at risk of RICH.

We investigated records for 68 patients who underwent HSCT during childhood or adolescence. Magnetic resonance (MR) imaging including T2^*^-weighted image of the brain was performed annually for 5 years, over a range of 6 to 29 years after HSCT.

Furthermore, we developed a scoring and grading system for CH in order to monitor the process and the progress of radiological changes. Among the 68 patients, 28 (41.2%) were diagnosed as CH.

All CH cases received total body irradiation as a conditioning treatment for HSCT and/or cranial radiation therapy prior to HSCT as part of the treatment for their primary disease. CH was found in none of those without radiation (*N*=19), in 46.2% of those who received 6–12 Gy (*N*=39) and in all of those who received 18–36 Gy (*N*=10). Total CH scores were correlated with higher radiation doses. Careful and long-term evaluation with MR imaging including T2^*^-weighted imaging is necessary for HSCT recipients who received radiation therapy prior to and/or during HSCT.

# Sub article: Problems in chimerism analysis using buccal mucosa after hematopoietic stem cell transplantation

**Published:** 2012-05-23

**Authors:** Osamu Hyodo, Humiko Tsuchida, Tatsuya Sugimoto, Kaoru Sato, Hiromasa Yabe, Fumiaki Yoshiba, Shunichi Kato

**Affiliations:** Department of Cell Transplantation and Regenerative Medicine, Tokai University School of Medicine, Isehara, Japan

## Purpose and Background

Chimerism analysis is important for evaluation of engraftment after hematopoietic stem cell transplantation (HSCT). Our department provides routine examination of chimerism by short tandem repeat (STR).

Oral buccal swab specimen is used as the host DNA sample source when pretransplant blood or bone marrow samples of the patient are absent. They are used because of the safety and the technical easiness to extract DNA.

However, these specimens do not always show 100% of host type STR. We investigated the reason of donor DNA contamination to the host buccal swab specimen.

## Materials and Methods

Buccal swab specimens before and after gurgle and saliva were collected from 40 HSCT recipients together with blood samples. Chimerism was analyzed using STR, which could recognize the difference between the donor and the recipient.

## Results

1. Blood samples from 37 out of 40 patients showed 100% donor type of STR, while stable mixed chimerism was recognized in the remaining 3 patients. 2. Buccal swab specimens before gurgle showed 16% of donor type STR in average, and the percentage of donor STR decreased by 7% after gurgle (*N*=20). 3. Saliva showed 44% of donor type STR in average (*N*=17).

## Conclusion

These results indicated donor type STR found in buccal swab specimens may be derived from the saliva to which donor cell the components were secreted.

# Sub article: Characteristic of *Candida* from fungoid-bacterial associations at inflammatory disorders of the upper respiratory tract

**Published:** 2012-05-23

**Authors:** Anatoliy P. Godovalov, Liliya P. Bykova, Anatoliy I. Morgunenko, Sergey Gris

**Affiliations:** 1 Acad. E.A. Wagner Perm State Medical Academy, Perm, Russia; 2 Medical Unit of Municipal Department of Internal Affairs on Perm region, Perm, Russia

In recent years, the world has seen an increase in the number of diseases caused by fungi. According to the World Health Organization, they suffer every fourth inhabitant of the planet, regardless of age, among the many varieties of fungi in the lead Candida. According to European Research, the presence of fungi on the surface of any body or organ is abnormal and should be prevented and treated intensively. These fungi can easily take root and take already populated by bacteria habitats. Of great interest are the features of their existence in microbiocenosis upper respiratory tract and connection between microbes with the action of antibiotics.

In this regard, the purpose of work was characterization of fungi of the genus *Candida*, isolated from the fungal–bacterial association of patients with inflammatory diseases of upper respiratory tract. Isolation of microorganisms was carried out using classical bacteriological method to the identification of species grown on cultural and biochemical characteristics. Determination of the sensitivity of microorganisms to the antimicrobial was performed using a disc-diffusion method. Adhesive properties of *Candida* were studied by the ability of cells of fungi attach to buccal epithelial cells. Adhesion strength tested by centrifugation.

In the study of 389 samples from the discharge of patients with inflammatory diseases of upper respiratory tract, fungi of the genus *Candida* were found in 15.2% of cases, and in the amount of 10^5^ colony-forming unit in 1 milliliter (CFU/ml) in 5.6%. Species spectrum of *Candida* was presented in 59.1% of *C. albicans*, in 18.2% of *C. pseudotropicalis*, in 13.6% of *C. krusei*, to 4.5% of *C. quilliermondii*. In a monoculture of *Candida*, fungi were found in 27.3% of samples. Among bacterial flora *Candida* fungi were often accompanied by Gram-positive cocci (85.7% of samples). Streptococci were found out in 83.3% of this samples. Association of *Candida* and gram-negative bacteria were found in 14.3% of cases (only members of the genus *Klebsiella*). Fungi of the genus *Candida* in association with staphylococci were susceptible to three antibiotics in all cases, and in association with streptococci—were susceptible to three antibiotics in 50%, 25%—they were sensitive to the two drugs and 25%—are only sensitive to one. All *Candida*, isolated in association with *Klebsiella*, were sensitive to three drugs.

In studying the adhesive properties, the factor of adhesion was 94.6±0.99%. Epithelial cells, free *Candida* cells were 5.40±0.99%. The average index of adhesion was 2.51±0.08 unit and microbial load, 57.2±2.55 unit. After centrifugation, the studied parameters were significantly decreased. The factor of adhesion was 54±2.21%, the average index of adhesion, 1.35±0.04 unit and microbial load, 11.05±1.24 unit (*p*<0.05 for all presented parameters before and after centrifugation). Studies have shown that strains of *Candida*, isolated in inflammatory diseases of upper respiratory tract, have middle adhesive properties, and further study of the adhesive ability to buccal epithelium reveals the true adhesive properties and eliminate the random adsorption.

Thus, it was found that the adhesive properties of fungi *Candida*, causing inflammatory diseases of upper respiratory tract, are of medium severity, and the mechanical impact on their adhesion to buccal epithelium does not alter the virulence properties of yeast-like fungi. Stability of the fungus *Candida* to antifungal drugs varies in microbial associations. The greatest stability of the genus *Candida* is registered in association with species of the genus *Streptococcus*. Latest in this case are sensitive to most antibiotics.

# Sub article: The role of sugars in the minor correction of immune status of beings

**Published:** 2012-05-23

**Authors:** Olga Sergeevna Korneeva, I.V. Cheremuskina

**Affiliations:** Voronezh State Technological Academy, Russia

The formation of the immune response is significantly affected by normal flora of the gastrointestinal tract. One of the ways to maintain is the use of prebiotics. Substances with prebiotic activity, being not digested carbohydrates in an unmodified form, easily reach the colon, where they become the promoters of normal flora. There exists an evidence that prebiotics can modulate various properties of the immune system, including the properties of lymphoid tissue. The most common factors are bifidogenic amino and neutral sugars, which include mannose and fucose.

An evaluation of prebiotic activity in *in vitro* tests revealed a tendency of positive effect of various concentrations of mannose, fucose and oligosaccharides on the growth of bifidobacteria. Marked by the ability of mannose- and fucose-containing components to increase the resistance of bifidobacteria cells in the presence of broad spectrum antibiotic, as well as the ability to increase symbiotic relationship between *B. bifidum* and *E. coli*, confirm their prebiotic properties.

The bifidogenic and lactogenic activity of mannose and manno-oligosaccharides was revealed in *in vivo* experiments. Their application in experimental dysbiosis, led to increasing of the number of bifidobacteria and stimulated the growth of lactic acid bacteria.

Studies of immune status of experimental animals, when introduced into their diet mannose-contained components, revealed a tendency for the positive effect of mannose to indicators of nonspecific immunity of experimental animals. Oral consumption of mannose and manno-oligosaccharides has a stimulating effect on the functional activity of macrophages, increasing absorptive capacity of the cells and digested. It was found a significant increase in activity of neutrophils in an experimental dysbiosis relatively intact animals, as well as the ability to induce the expression of mannose pro- and anti-inflammatory cytokines.

The introduction of fucose in the diet of mammals has a positive effect on the degree of oocyte membrane fucosilation and reproduction of experimental animals.

Studies of immunoglobulins synthesized with different carbohydrate diet showed that the qualitative composition of immunoglobulins is significantly changed after the application of mannose diet. The introduction of fucose to the diet of mammals provided the stimulating effect on antibody production.

Taking into account all the data, there is no doubt that an effective supplement on the basis of mannose and fucose-containing components will be a prerequisite for the restoration of biochemical processes in the body and improve the immune status.

The research was done under the Federal target program ‘Research and pedagogical staff or innovative of Russia’ (SC Nos. P260 and P1074).

# Sub article: Characteristics of interaction between tick-born encephalitis virus and human immune system

**Published:** 2012-05-23

**Authors:** Liliya P. Bykova, Rafael Z. Kuziaev, Anatoliy P. Godovalov, Sergey Gris

**Affiliations:** 1 Acad. E.A. Wagner Perm State Medical Academy, Perm, Russia; 2 Medical Unit of Municipal Department of Internal Affairs on Perm region, Perm, Russia

The tick-borne encephalitis is the neurovirus infection, which is widespread on the Western Ural territory, the natural center of tick-borne infections. The tick-borne encephalitis infection develops owing to transmissible or alimentary introduction of neurotropic virus and its interaction with a human body. This interaction is defined by properties of the originator and reactance of a macroorganism. Many researchers believe that tick-borne encephalitis severity depends on its ability to get through a blood-brain barrier with brain lesion or without it. The nervous system lesion is characterized by polymorphism of implications. Feature of the infection pathogenesis is the ability of tick-borne encephalitis virus to remain active for a long time, even against treatment of this disease. According to clinical data, frequency of infection transformation into the chronic form is 3–11% to total number of cases of acute type disease. The immune response begins with activity of innate immunity factors, including phagocytosis. Further virus neutralization is connected with the activity of antibodies and specific cellular factors. Period of virus conservation in an organism and a condition of patient's immune system are of interest.

The research objective is to define RNA of the tick-borne encephalitis virus and to study the cellular and humoral immune response of the patient with chronic form of disease.

We observed 33 people with different disease terms and clinical implications of chronic tick-borne encephalitis. The blood and serums received from patients were research material. We used PCR to define RNA of the virus and ELISA to define immunoglobulins of M and G classes. Assessment of phagocytic activity of blood neutrophils was carried out by a method with formalinized sheep erythrocytes.

Researchers have found that 60% of patients had RNA of the tick-borne encephalitis virus in their blood. Thus, disease terms varied from 1 to 15 years. Phagocytic reaction was shown by raised activity and made 65.4±4.1%. Study of phagocytic reaction in the presence of specific viral antigen showed a negligible increase in the activity of phagocytosis (*p*>0.05). 70% of examined people had immunoglobulins. People with RNA of the virus had IgM in 57% of cases and IgG in 40% (Fig. 1). Research studies have taped ineffective activation of a cellular and humoral link of the immune response in chronic form of tick-borne encephalitis. Detection of RNA of the tick-borne encephalitis virus in late disease terms shows the possibility of long persistence of the virus. It can be persistent during humoral immune response but it is ineffective in protection against the tick-borne encephalitis virus. The inefficiency of phagocytic response is possibly connected with the fact that the virus damages phagocytic system. These are the mechanisms allowing the virus to escape action not only of humoral protection but also of cellular one. Fluctuations of researchers’ results can be connected with specific features of the patients’ immune status and strain virus characteristics. PCR could be included into a complex of methods for studying tick-borne encephalitis as an additional way to diagnose chronic tick-borne encephalitis.

**Fig. 1:** The amount of specific immunoglobulins M and G to the virus (geometric titer) and the number of tick-borne encephalitis patients in whom viral RNA was found (%).

# Sub article: Changes in phenotypic and functional properties of mononuclear leukocytes as a result of bacterial vaccine application under induced immunosuppression

**Published:** 2012-05-23

**Authors:** Olga V. Lebedinskaya, Elena A. Lebedinskaya, Nelly K. Akhmatova, Anatoliy P. Godovalov, Sergey Gris

**Affiliations:** 1 E.A. Wagner Perm State Medical Academy, Perm, Russia; 2 I.I. Mechnikov Scientific Research Institute of Vaccines and Sera, RAMS, Moscow, Russia

Treatment of malignant tumors is accompanied by the suppressive action on the immune system that is related to numerous factors and chemotherapeutic preparations in particular. Examination of immunological parameters while using cytostatics is attractive for the purpose of searching an adequate method of correction of subsequent alterations. The aim of work was the study of the effect of bacterial vaccine Immunovac (VP-4) on the immune phenotype and the functional activity of murine spleen lymphocytes against a background of cyclophosphane (CP)-induced immunosuppression. Investigations involved the use of preparations: CP and multicomponent vaccine Immunovac (I.I. Mechnikov SRIVS, PAMS, Moscow) that is composed of antigenic components of *Staphylococcus aureus*, *Klebsiella pneumoniae, Proteus vulgaris* and *Escherichia coli*. Alongside with its typical neutropenic action, CP being introduced into mice provoked marked immunosuppression that was manifested in significant lowering in absolute lymphocyte number in murine peripheral blood and spleen. Lymphopenia was primarily expressed in the reduction of helper lymphocytes (CD4^+^), B-cells and NK-cells (Fig. 1). In addition, CP immunosuppressive action was demonstrated via decrease in the NK-cell functional capability that was not recovered even by the fifth day of the experiment (Table 1).

**Fig. 1:** The expression of surface molecules CD19/CD8, CD25/CD4 and DX5/CD3 on mice spleen mononuclear leukocytes. Double vital staining with monoclonal antibodies.

**Table 1:** Effect of VP-4 on NK-activity of mice spleen cells against CP-induced immunodeficiency

			NK-activity at a ratio—the target cell/effector		
			
Time after administration, h	Group number	Preparation	1:05	1:02	1:01
0	1		73.4±5.6	39.3±1.2	27.9±0.8
48	2	CP	34.1±3.7*	20.1±2.2*	14.5±0.6*
	3	CP + VP-4	63.2±2.9**	40.8±2.7**	22.3±1.2**
96	4	CP	39.6±1.2*	27.1±3.1*	9.3±0.1*
	5	CP + VP-4	57.3±2.4**	35.4±1.8	12.9±0.1*
168	6	CP	52.7±3.1*	43.6±0.9	35.4±1.5
	7	CP + VP-4	49.5±1.9*	41.4±2.0	33.9±2.7

* *p*<0.05 compared to control.

** *p*<0.05 compared to CP.

Such alterations could result in insufficiency of innate and adaptive components of immunity. Native immune modulator VP-4 was found to level the CP-induced lymphopenia, as well as alterations in lymphocyte subpopulation composition and their functional state.

The advantage of combined application of CP and given immune modulating preparation appears to be faster approaching the normalizing effect as compared with the cytostatic action. This fact seems to be crucial for prevention of infectious complications occurring in cancer patients with chemopreparation-induced immunosuppression. Considering earlier observations of the VP-4 ability to promote anti-infective and antitumor immunity, it seems expedient to carry out clinical trials of this preparation for the prevention of the immune status disorders in cancer patients after the chemotherapy treatment. This work was supported by RFBR Grant №11-04-96037 rural and administrative body of Perm Region.

# Sub article: Anti-inflammatory properties of *Bifidobacterium* spp. strains isolated from healthy infant feces

**Published:** 2012-05-23

**Authors:** Ekaterina V. Khokhlova, Boris A. Efimov, Lyudmila I. Kafarskaik, Svetalana I. Pavlova, Andrei N. Shkoporov

**Affiliations:** 1 Department of Microbiology and Virology, Russian State Medical University, Moscow, Russian Federation; 2 Department of Pharmacology, Russian State Medical University, Moscow, Russian Federation

Several groups have reported recently that certain probiotic *Bifidobacterium* strains are able to inhibit inflammatory response in intestinal epithelial cells *in vitro* and *in vivo*. However, neither chemical nature nor precise mechanism of action of putative anti-inflammatory agents has been determined. In addition, it is unclear whether such immunomodulating activity is a property of some specific *Bifidobacterium* strains or is common for the whole genus. Here, we report on partial characterization of similar anti-inflammatory properties in several *Bifidobacterium* strains, isolated from healthy infant feces. It was found that conditioned media (CM) of all tested strains was capable to attenuate, albeit to varying extent, TNFα- and LPS-induced inflammatory response in HT-29 colon epithelial cell line. In contrast, neither killed bifidobacterial cells nor cell extracts possess such activities. The active substance present in bifidobacterial CM was found to be resistant to protease and nuclease treatment, heat-labile, nonlipophilic and having molecular weight of less than 3 kDa. The anti-inflammatory effect of bifidobacterial culture supernatants was dose- and time-dependent and was accompanied by inhibition of IκB phosphorylation and NF-κB-dependent promoter activation. *Bifidobacterium* CM were found to be nontoxic to HT-29 cells and did not influence gene expression pattern in the absence of proinflammatory stimuli. However, combined treatment of cells with CM and LPS or TNFα resulted in upregulation of TGFβ1, IκBκ and p21^CIP^ mRNAs.

# Sub article: Physiological changes of liver induced by fluoroquinolones and hepatoprotective effect of CAP18/LL-37 synthetic peptide in mice

**Published:** 2012-05-23

**Authors:** Shiaki Takagi, Yoko Sugawara, Katsuya Inada, Hiroshi Isogai, Emiko Isogai

**Affiliations:** 1 Department of Animal Microbiology, Graduate School of Agricultural Science, Tohoku University, Sendai, Japan; 2 Iwate University, Iwate, Japan; 3 Division of Animal Experimentation, Sapporo Medical University, Hokkaido, Japan

## Objectives

It has been reported that patients with severe bacterial diarrhea sometimes showed hepatic dysfunction after antibiotic treatment. Antibiotic treatment is also reported to induce lipopolysaccharide (LPS) from the infectious bacteria. The upregulation of TNF-alpha expression has been reported as pathogenic symptoms in human hepatitis. In this study, we examined whether hepatic physiological changes related to LPS release and TNF-alpha production occurs *in vivo* when fluoroquinolones were administered to SPF mice with intestinal flora. We also examined the hepatoprotective effect of CAP18/LL-37 synthetic analogue peptide, which has strong LPS-binding activity, on LPS-inoculated mice.

## Methods

Sitafloxacin (STFX) and levofloxacin (LVFX) were used as antibiotics. ICR mice (SPF, male, 5-weeks-old) were used. In the first experiment, the antibiotics (5 or 20 mg/kg/day) were administered to the mice once a day for 5 days intraoral or abdominal administration. In the second experiment, LPS (*Shigella flexneri* serotype 1A) was administered 1 h after the administration of the peptide for neutralization of LPS. In both experiments, blood, liver and spleen were collected from the mice. The concentration of ATL, AST, TNF and LPS were measured.

## Results

In the first experiment, ALT and AST increased after antibiotic administration. LPS in the blood plasma was detected from 1 to -8 days after STFX or LVFX. TNF-alpha was detected in the plasma from the mice treated with STFX and in liver from the mice treated with STFX (or LVFX). On eighth day, TNF-alpha was not detected in any mice. In second experiment, ALT and AST increased after inoculation of LPS but not in the mice treated with the synthetic peptide. TNF-alpha also did not detect in the peptide treated mice.

## Conclusion

We showed increase in AST and ALT after treatment of STFX or LVFX in normal SPF mice. CAP18/LL-37 synthetic analogue peptide can protect mice/human from the released-LPS after antibiotic administration.

# Sub article: Role of IL-10 and IL-17 in the *Mycoplasma pneumoniae* experimental pneumonia model

**Published:** 2012-05-23

**Authors:** Satoshi Kurata, Haruhiko Taguchi, Takako Osaki, Tomoko Hanawa, Hideo Yonezawa, Shigeru Kamiya

**Affiliations:** 1 Department of Infectious Diseases, Kyorin University School of Medicine; 2 Department of Immunology, Kyorin University Faculty of Health Sciences, Tokyo, Japan

*Mycoplasma pneumoniae* is a common cause of community-acquired respiratory tract infection, and the participation of the excessive reply of the host immunity is thought to be involved in mycoplasmal pneumonia and the onset pathogenic mechanisms of the various complications. Recently, several studies showed that Th17 cells as the third positive effector T cell, characterized by production of IL-17, IL-21 and IL-22 have been implicated in the onset of autoimmune diseases. On the other hand, Treg cells have been reported to contribute to suppress a hyper immunoresponse, and T cell subsets except Th1 and Th2 cells were inferred to be involved in pathogenic mechanisms of mycoplasmal pneumonia and the complications.

In this study, we established an experimental pneumonia mouse model by the use of *M. pneumoniae* sonicated crude antigens and performed pathological and immunological analyses to examine the induction mechanisms of Th17 cells. Additionally, we have examined the specificity of Th17 cell inducibility by using mouse splenocytes. *In vivo* analysis indicates that mRNA expression levels of IL-17 and IL-10 in the lung were increased by the high concentration and high frequency of *M. pneumoniae* antigens sensitization. *M. pneumoniae* antigens sensitization caused the lung inflammation with infiltration of neutrophils and macrophages. *In vitro* analysis indicates that *M. pneumoniae* antigens induced IL-17 release from mouse splenocytes in a concentration-dependent manner.

These results suggested that effective sensitization of *M. pneumoniae* antigens induced Th17 cells differentiation, and this immnoresponse was suppressed by Treg cells producing IL-10.

# Sub article: Wild deer have antibodies recognizing tick defensin with tertiary structure

**Published:** 2012-05-23

**Authors:** Emiko Isogai, Nayuta Isogai, Kazuki Sato, Hiroshi Yoneyama, Tomoichi Fukuda, Yoshitake Deguchi, Kazuei Matsubara, Hideo Murata, Takamitsu Tsuboi, Makoto Haritani, Toru Miyamoto, Hiroshi Isogai

**Affiliations:** 1 Department of Animal Microbiology, Graduate School of Agricultural Science, Tohoku University, Sendai, Japan; 2 Division of Animal Experimentation, Sapporo Medical University, Hokkaidō, Japan; 3 Faculty of Agriculture, Iwate University, Iwate, Japan; 4 Faculty of Life Science, Kyoto Sangyo University, Kyoto, Japan; 5 National Institute of Animal Health, Ibaraki, Japan

## Objectives

*Borrelia burgdorferi, B. garinii* and others, the aetiological agents of the tick-borne zoonosis Lyme disease, circulate between ticks and vertebrate hosts. In the transmission and maintenance cycle of the spirochetes, it is important to know the density of vector ticks. After tick bite, antibodies against Lyme disease spirochetes have been detected in human and animals. Vector competence for Lyme disease spirochetes has been confirmed for tick species. Tick defensins, small cysteine-rich cationic proteins, are active against various bacteria but not against Lyme disease spirochetes. Therefore, we examined antibodies against tick defensins in the sera from wild deer in different ecology (with different tick species). The importance of the tertiary structure of tick defensin in antibody recognition in wild deer was also examined.

## Methods

Blood of the wild sika deer was collected from three distinct areas, Hokkaido, Aomori and Iwate, in Japan. We synthesized the tertiary and linear peptides on the information of the sequence in defensin originated from the taiga tick, *Ixodes persulcatus*. Antibody detection was done by ELISA.

## Results

Both tertiary and linear synthetic peptides reacted with antibodies from wild deer. The antibody titer against tick defensin with tertiary structure was significantly higher than that against linear one. The antibodies from wild sika deer recognized the tertiary structure. Tick defensin shows different amino acid sequence. In Hokkaido, 98.8% of sika deer sera are positive against tick defensin from *I. persulcatus*. The positive percent of the deer in Aomori and Iwate was significantly lower than that in Hokkaido (major habitat: *I. persulcatus*). In Aomori and Iwate, deer has antibodies against *Haemaphysalis longicornis*. No antibodies against *Ornithodoros moubata* were detected in deer.

## Conclusion

The tertiary structure of tick defensin is important to recognition in antibody response in wild deer. We can know vector potential by detection of antibodies against the tick-species specific defensin. The wild animal monitoring system is useful to get information of zoonotic diseases such as Lyme disease.

# Sub article: Gut indigenous microbiota and epigenetics

**Published:** 2012-05-23

**Authors:** Boris A. Shenderov

**Affiliations:** Moscow Research Institute of Epidemiology and Microbiology after G.N. Gabrichevsky, Moscow, Russia

Any phenotype is the result of complex interactions between genotype, epigenome and environment. Epigenetics focuses on processes that regulate how and when certain genes are turned on and turned off, while epigenomics pertains to analysis of epigenetic changes across many genes in a cell or entire organism. The epigenome combines molecular events that integrate genotype with phenotype. Epigenetics is defined as gene-regulating activities that do not connect with alteration of the base sequence. In recent years, it has become increasingly clear that epigenetic regulation of gene expression is critical during all stages of living being life, and perturbations in the epigenetic mechanisms can result in mammalian health disturbances. Epigenomic effects are connected with the covalent attachment of different chemical groups to DNA, histones, chromatin and to different other associated proteins during posttranslation period. Biochemical basis of epigenetics are methylation, acetylation, phosphorylation, ubiquitylation, ADP-ribosylation, biotinilation, sumoyalation, repeat-induced gene silencing, mircoRNA interferences and some other processes. Epigenomic reprogramming of cell genome and posttranslation modification of gene expression are essential mechanisms of the development, regeneration and postnatal life of higher eukaryotic organisms (gene expression regulation, cell proliferation, cellular stress events, aging and DNA repair, lifelong circadian drifts, equilibrium between mitosis and apoptosis, modification of bacterial and host cell quorum sensing, host–bacteria cross-talk and so on). All these epigenetic modifications can be causally involved in a broad spectrum of human condition including various monogenic and multifactorial disorders (metabolic syndrome, type II diabetes, schizophrenia, autoimmune diseases, cancer, autism and so on). Epigenetics may help explain some features of complex diseases such as late onset, gender effects, fluctuation of symptoms, phenotypic differences between monozygotic twins and others. Epigenetic code can be individual, tissue and cellspecific and may change over time because of aging, disease or environmental factors (e.g., nutrition, life style and toxin exposure).

However, the epigenetic reprogramming and posttranslated mechanisms remain unclear in details. The mammalian gastrointestinal tract is colonized by the vast majority of symbiotic microorganisms. Indigenous microbiota has the intriguing diversity and produce extremely important contributions to human physiology, biochemistry and behavior. In the recent years, it has been established indigenous microbiota produce multiple low molecular weight (LMW) substances (lacton-like autoinducers, peptide pheromones, protein autoinducers, stress proteins, SCFA and other organic acids, various enzymes, amino and nucleic acids, nucleotides, vitamins, amines, polyamines, hormone-similar substances, polysaccharides, oligosaccharides, peptidoglycans, lipoteichoic acid, glycopeptides, lipopolysaccharides, antimicrobial compounds, lectins, biosurfactants and many others), which can quickly be distributed along human organisms and interact with different targets in the host cells and tissues. These LMW compounds are considered to be potential autoinducers regulating prokaryotic and eukaryotic growth and development, bacteria–bacteria and host–bacteria relationships. Recent genomics-, epigenomics- and metabolomics-based studies have permitted to provide insights into how indigenous microbiota (including probiotics) sense and adapt to the gastrointestinal tract environment and regulate gene expression and posttranslation modification of gene determined final products in prokaryotic and eukaryotic cells in and outside host intestinal tract. It is known that gene expression governs human existence. Above-mentioned microbial LMW compounds may be one of the key endogenous environmental factors to which human genes are exposed throughout life. Indigenous microbiota, in turn, governs the concentration of different eukaryotic cell producing proteins in different organs by functioning as regulators of stability DNA and RNA, gene transcription and translation, RNA processing, mRNA degradation, posttranslation modification and so on. The intensity of microbial signal and the subsequent response can vary with the composition and number of certain intestinal microorganisms. The physiological condition of the individual also may determine which human genes are influenced by indigenous gut microbiota that can serve both as source of substrates, cofactors or coenzymes for different metabolic processes and as a donor of various chemical groups (methyl-, acetyl-, phosphate-, etc), modified bases, signaling molecules or enzymes participating in the processes connected with epigenomic regulation, co-factors of these enzymes and as a donor of microbially generated metabolites from certain food components. All of them are able to interfere in the host metabolism, genomic and epigenomic processes. Besides, some LMW molecules of microbial origin can activate or inhibit epigenomic regulation through interfere to activity of enzymes (methyltransferases, deacetylases, acetyltransferases, phosphotransferases, kinases and so on) that are participants in epigenomic reprogramming and/or posttranslation modification histone and other proteins. LMW substances and metabolites produced by pregnant woman gut microbiota can penetrate to fetus resulting in permanent effects its development programming, cognitive function, metabolism and body composition in the natal and postnatal periods of life through activation or suppression of gene expression, or turning genes ‘on’ or ‘off’. Integration of genomics, epigenomics, nutrition and microbiota permits to understand how LMW molecules of diet and microbe origin effect gene expression and posttranslation modification. These observations can become the basis for development of novel approaches in designing new medicines and diagnostic tests for different pathological syndromes having epigenetic components.

The author substantiates the necessity to create International Project ‘Human Gut Microbiota and Epigenetics’. The goals of this project will be to facilitate interdisciplinary collaborations among scientists engaged in host microbial ecology, metagenomics, epigenomics, nutrition, health support, diseases prevention and treatment researches. The main goal of such investigations will be the establishment of the profile of microbe-connected LMW substances, which characterize the role of human indigenous microbiota in the epigenomic regulation of genome and microbiome activity and protein posttranslation modification. Midterm goal is to provide metabolic databases that could be used for selection of microbe-associated biologically active LMW molecules participating in the epigenomic processes relevant to human health and metabolic diseases.

# Sub article: Impact of dihydrodaidzein-producing *Clostridium*-like intestinal bacterium, strain TM-40 on *in vitro* metabolism of daidzein by equol-producing bacterium and fecal microbiota from human

**Published:** 2012-05-23

**Authors:** Motoi Tamura, Sachiko Hori, Yukie Kurusu, Hiroyuki Nakagawa

**Affiliations:** National Food Research Institute, National Agriculture and Food Research Organization, Tsukuba, Japan

Much attention has been focused on the biological effects of equol, a metabolite of daidzein produced by intestinal microbiota. However, little is known about the role of isoflavone metabolizing bacteria in the intestinal microbiota. Recently, we isolated a dihydrodaidzein (DHD)-producing *Clostridium*-like bacterium, strain TM-40, from human feces. We investigated the effects of strain TM-40 on *in vitro* daidzein metabolism by equol-producing bacterium TM, human fecal microbiota from a male equol producer and two male equol nonproducers. Adding strain TM-40 to the incubation solution of equol-producing strain TM greatly increased equol production from daidzein *in vitro*. In the fecal suspension from the male equol nonproducer and DHD producer, DHD was detected in the *in vitro* fecal incubation of daidzein by adding TM-40. The DHD concentration increased as the concentration of strain TM-40 increased. In the fecal suspension from the equol producer, the fecal equol production was increased by adding strain TM-40. The occupation ratios of *Bifidobacterium* and *Lactobacillales* were higher in the equol nonproducers than in the equol producer. Adding isoflavone-metabolizing bacteria to the equol-producing bacterium or fecal microbiota used in our investigation should facilitate the estimation of the metabolism of the isoflavonoids by the fecal microbiota.

Studies on the interactions between the equol-producing microbiota and DHD-producing bacteria might lead to clarification of some regulating mechanisms of equol production by fecal microbiota.

# Sub article: Microflora of pregnant women and methods of its correction at disbiosysis

**Published:** 2012-05-23

**Authors:** Taras M. Lyaskovsky

**Affiliations:** Institute of Microbiology and Virology, National Academy of Sciences of Ukraine, Kiev, MSP, Ukraine

**Keywords:** Lactic acid bacteria, identification, antagonistic properties, adhesive activity, antibiotic sensitivity, interferonogenic, antivirus activity of probiotic strains

Quantitative and genus stuff of vaginal microflora and intestine of pregnant women have been examined. At the result of the study, main taxonomic groups of microorganisms present in the vaginal canal and in the intestine have been isolated. Main pathogens, which cause microflora disbalance and as a result provoke vaginal disease, were examined. They are mainly represented by *Gardnerella vaginalis*, *Escherichia coli*, anaerobic bacteria, *Candida* sp. and so on.

Lactic acid bacteria were represented by *Lactobacillus acidophilus*, *L*. *plantarum*, *L. fermentum*, *L. vaginalis*; *Enterococcus faecium*, *E. faecalis*, *E. durans; Streptococcus thermophilus* and *Lactococcus lactis*.

Identification of probiotic strains of lactic acid bacteria based on phenotype characteristics (API-testing) and molecular biology techniques with the use of specific primers confirmed the genus of *S. thermophilus*, *E. faecium* and *L. plantarum*.

The antagonistic properties of probiotic cultures of lactic acid bacteria toward referent strains of pathogenic microorganisms have been shown. Antagonistic activity depends on the genus and strain of lactobacilli as well as conditions of cultivations. Adhesive activity of probiotic strains of lactic acid bacteria to vaginal epithelium has also been shown. For the first time, the change of antibiotics sensitivity of lactic acid bacteria on the model of lymphoblastoid cells has been indicated. The ability of probiotic strains to induce alfa and gamma interferons, as well as to inhibit flu and genital herpes viruses activity, has been exhibited. Probiotic substances have been proposed for the purpose of use as prophylactic and curing preparations for vaginal and intestinal dysbacteriosis treatment.

# Sub article: Comparative analysis of gastric bacterial microbiota in Mongolian gerbil after long-term infection with *Helicobacter pylori*

**Published:** 2012-05-23

**Authors:** Takako Osaki, Cynthia Zaman, Takahiro Matsuki, Takashi Asahara, Hideo Yonezawa, Tomoko Hanawa, Satoshi Kurata, Fuhito Hojo, Haruhiko Taguchi, Shigeru Kamiya

**Affiliations:** 1 Department of Infectious Diseases, Kyorin University School of Medicine, Japan; 2 Yakult Central Institute for Microbiological Research, Yaho Kunitachi Tokyo, Japan; 3 Department of Immunology, Kyorin University Faculty of Health Sciences, Japan

The quantitative (qt) real-time PCR using 16SrDNA primers is useful for determination of bacterial composition in Mongolian gerbil's gastric microbiota. The aim of the present study was to determine the differences of the gastric microbiota after long-term infection with *Helicobacter pylori*. At after 1 year inoculation with *H. pylori*, five gerbils were determined as *H. pylori*-positive and 6 gerbils were *H. pylori*-negative by culture and real-time qt PCR methods. In the gastric mucus of Mongolian gerbils, DNA of *Atopobium* cluster, *Bifidobacterium* spp., *Clostridium coccoides* group, *Clostridium leptum* subgroup, *Eubacterium cylindroides* group, *Enterococcus* spp., *Lactobacillus* spp. and *Prevotella* spp. were detected. The numbers of *C. leptum* subgroup, *C. coccoides* group and *Bifidobacterium* spp. in gastric mucus of *H. pylori*-negative Mongolian gerbils were significantly lower than those in noninfected gerbils and *E. cylinderoides* group and *Prevotella* spp. were detected only in *H. pylori*-negative gerbils. The composition of the gastric indigenous microbiota in the Mongolian gerbil was disturbed by long-term infection with *H. pylori*, and the existence of *H. pylori* might be also inhibited by the changed microbiota.

# Sub article: Gut colonization shapes the host's metabolism

**Published:** 2012-05-23

**Authors:** Sandrine P. Claus, Léa Maître, Steve Robinette, Sunil Kochhar, Jeremy K. Nicholson

**Affiliations:** 1 Department of Food and Nutritional Sciences, The University of Reading. Whiteknights, PO Box 226, RG6 6AP, Reading, United Kingdom; 2 Biomolecular Medicine, Department of Surgery and Cancer, Faculty of Medicine, Imperial College London, SW7 2AZ, London, United Kingdom; 3 Nestlé Research Centre, NESTEC Limited, Vers-chez-les-Blancs, Lausanne, Switzerland

Gut microbiota are an essential component of the host metabolism, providing nutrients as well as stimulating the host immune system. This symbiosis is the consequence of a long co-evolution, which results in a relatively low level of diversity compared to most soil ecosystems. This implies that the host adapts its metabolism to the presence of microbial symbionts, an adjustment that starts at birth in parallel to the beginning of gut colonization. Simultaneously to this major event, many fundamental modifications occur such as immune development and brain maturation.

In a recent study, we characterized the metabolic phenotype of germfree mice by a ^1^H NMR-based metabonomic approach coupled with pattern recognition methods (1). We further examined the global effects of gut colonization on the host metabolism in the same germfree mouse model monitored during 20 days (*n*=35/group, 5 time-points). Acquisition of the gut microbiota was associated with rapid increase in body weight (4%) over the first 5 days of colonization with parallel changes in multiple pathways in all compartments analyzed (i.e., urine, plasma, liver, kidney and colon). The colonization process stimulated glycogenesis in the liver prior to triggering increased in hepatic triglyceride synthesis. These changes were associated with modifications of hepatic Cyp8b1 expression and the subsequent alteration of bile acid metabolites, including taurocholate and tauromuricholate, which are key regulators of the global hydrophobicity of the bile acid pool and consequently control cholesterol and lipid absorption. Activity and expression of the drug metabolizing enzymes Cyp3a11 and Cyp2c29 were also significantly altered in germfree animals. Finally, correlations between the hepatic metabotype and microbiota composition analyzed by 16S-pyrosequencing revealed strong associations of the *Coriobacteriaceae* (genus *Eggerthella)* with the hepatic triglyceride levels, energy metabolites and Cyp3a11 activity, the equivalent of the human CYP3A4, a major constituent of drug mono-oxygenation (2).

We then extended this study by analyzing the brain metabolite signature over the colonization process using^1^H high resolution magic angle spinning NMR spectroscopy concomitantly with the ^1^H NMR metabolic profile of fecal waters in the same animals. The metabolic profiling of fecal water at several time-points revealed the rapid appearance of SCFAs (acetate followed by butyrate) concurrently with the disappearance of complex oligosaccharides as a result of their fermentation by colonic bacteria. Protein breakdown also occurred early as demonstrated by the rapid appearance of branched-chain amino acids (valine, leucine and isoleucine) and aromatic amino acids such as phenylalanine, tyrosine and histidine. Altogether, these results gave insights into the biochemical reactions that occurred in the intestine during gut microbial acquisition. Simultaneously, we observed a considerable impact of the lack of gut microbiota on brain metabolism in the two investigated sections (i.e., cerebellum and cortex). In particular, a remarkable increase in *scyllo*-inositol concentration was consistently observed in germfree animals. This was accompanied by higher levels of choline and aspartate in the germfree cortex and higher levels of fumarate and glutamate in the germfree cerebellum. All of these metabolic abnormalities were normalized 10 days postcolonization, indicating a rapid recovery of brain metabolism driven by the presence of gut bacteria. These results support recently published data highlighting differential gene expressions in the cortex and the cerebellum of germfree animals compared to their conventional counterparts (3).

Altogether, these results give new insights into the biochemical mechanisms associated to the multiple metabolic and behavioral perturbations reported in germfree animals. They also provide a basis to further develop novel strategies in the alteration of the host–microbiota relationship to beneficially modulate the host metabolism.

## References

[1] Claus SP, Tsang TM, Wang Y, Cloarec O, Skordi E, Martin F-P (2008). Systemic multicompartmental effects of the gut microbiome on mouse metabolic phenotypes. Mol Syst Biol.

[2] Claus SP, Ellero SL, Berger B, Krause L, Bruttin A, Molina J (2011). Colonization-induced host-gut microbial metabolic interaction. MBio.

[3] Heijtz RH, Wang S, Anuar F, Qian Y, Björkholm B, Samuelsson A (2011). Normal gut microbiota modulates brain development and behavior. Proc Natl Acad Sci USA.

# Sub article: Features of intestinal *microbial biocenoses* in dysbiotic individuals

**Published:** 2012-05-23

**Authors:** Elena F. Zavgorodnyaya, Lyudmila A. Stashkevich

**Affiliations:** Khabarovsk Research Institute of Epidemiology and Microbiology, Federal State Institution of Science under the Federal Service for Supervision in the Field of Consumer Right Protection and Human Welfare, Russian Federation

Incidence as well as certain biological properties of opportunistic pathogenic bacteria (OPB), isolated from microbial associations and monocultures of individuals with intestinal dysbioses, were analyzed.

Colonic microflora of 1,690 individuals, including 1,587 two-month- through three-year-old children and 103 adults, as well as their OPB isolates, was examined.

Dysbacteriosis was found in 98.8% of children and 93.2% of adults. An increase of OPB quantity to numbers exceeding an age-appropriate standard was responsible for dysbioses in 75.5% of children and 69.9% of adults. *Klebsiella* bacteria were prevalent in the structure of intestinal microbial biocenoses in both children and adults, accounting for 80.0% and 68.5%, respectively. *Staphylococcus aureus* accounted for 12.0% in children and 3.7% in adults. Other Enterobacteriaceae were much more infrequent and accounted for 9.7% of children's biocenoses and 11.1% of adults^,^ biocenoses.

*Candida* fungi were isolated from 65.6% of children and 34.4% of adults. In terms of species structure, *C. albicans* (61.9% of all fungi isolated from children and 54.6% isolated from adults) and *C. glabrata* (33.3% and 45.5%, respectively) were prevalent; *C. tropicalis* and *C. krusei* were isolated in much lesser quantity and from children only.

In recent years, the number of dysbioses caused by microbial associations has increased. Associations of various OPB with bacteria of genus *Klebsiella* are most common. Thus, said microorganisms were isolated from associations in 57.6% of children under 1 year of age, 49.3% of children under 2 years of age, 40.0% of children under 3 years of age and in 32.7% of adults. *Klebsiella*+*S. aureus* association was most common in children, accounting for 72.9% in children under 1 year of age, 46.4% in children under 2 years of age and 35.7% in children under 3 years of age. Said association was found in 30.0% of adults, while associations of *Klebsiella*+fungi of genus *Candida* were most common (55%). Hemolytic *E. coli* ranked second among *S. aureus*’ associates in all the groups examined (in 66.3% of children and 10.0% of adults, in the average), while, as a monoculture, it was isolated from 50.3% of all the children and 1.9% of all the adults examined.

It was discovered that *Klebsiella*+*S. aureus* association in 82.8% of individuals included *Klebsiella* strains of great hemolytic activity, while monocultures displayed hemolytic properties in 17.2% of cases.

About 18.8% of phage-sensitive strains were only isolated among *Klebsiella* organisms, which is due to significant occurrence of *K. oxytoca* in intestinal biocenoses. It should be emphasized that circulation of *K. oxytoca* strains tends to increase and now exceeds the number of *K. pneumoniae* strains isolated. 94.3% of phage-resistant *Klebsiella* strains were isolated from associations.

Among the strains of *S. aureus*, 27.4% were found to be resistant to staphylococcal and 42.5% to polyvalent pyobacteriophages. Associate strains were found to demonstrate much greater degree of resistance manifestation (85.0% and 78.0%, respectively).

The study of antagonistic activity of associate strains (*Klebsiella*+*S. aureus*) revealed a high degree of antagonistic activity. Thus, associate *Klebsiellae* yielded 76.7% of highly active strains isolated, while monocultures, 63.6%. Similar figures for *S. aureus* were 72.5% and 57.1%, respectively.

Thus, in dysbioses, OPB appear prevalent in microbial biocenosis of children and adults; isolation of these microorganisms from associations proves to be much more frequent; microbial association strains appear to have more marked antagonistic activity and phage-resistance than monocultures.

# Sub article: Evaluation of sterility tests for cord blood banking in the Tokai University Cord Blood Bank

**Published:** 2012-05-23

**Authors:** Tatsuya Sugimoto, Hitomi Sasaki, Rie Ando, Sachiyo Sakama, Chie Nakashioya, Fumiko Tsuchida, Kaoru Sato, Shunichi Kato

**Affiliations:** Tokai University Cord Blood Bank, Tokai University Hospital, Isehara, Japan

Cord blood has been increasingly used as the source of hematopoietic stem cell transplantation. Sterility test (ST) is important for quality assurance of cell products. However, the material and method for ST is not unified among 11 cord blood banks of the Japan Cord Blood Bank Network (JCBBN).

ST had been performed using only a part of the final product by BACT/ALERT culture previously in our bank, but a part of red cell concentration was added as material, and Oxioid SIGNAL Blood Culture System Medium (Oxioid method) was employed as method other than BACT/ALERT culture in August 2009.

Incidence of bacterial positivity was compared between October 2008–July 2009 (group 1) and August 2009–May 2010 (group 2). Bacterial positivity was 1.3% in group 1 and 3.0% in group 2 (*p*=0.052). Although the difference was marginally significant, some bacteria that could not be found in the previous method were identified by the newly employed method.

These results suggest that this new ST could contribute to the safer cord blood product.

# Sub article: The study of the bacterial contamination in the spray water of electronic toilets and in the gluteal and inguinal regions due to splashing following spray water

**Published:** 2012-05-23

**Authors:** Hideki Katano, Kumi Yokoyama, Yasushi Takei, Hideaki Matsuki, Mami Tsukiji, Seiki Tazume

**Affiliations:** 1 School of Health Sciences, Tokai University, Tokyo, Japan; 2 Venex Research Institute, Kanagawa, Japan; 3 Tokyo Healthcare University, Tokyo, Japan; 4 Gifu University of Medical Science, Seki, Japan; 5 Kanagawa Prefectural University of Human Service, Yokosuka (in original Yokohama), Japan

## Objective

The function of the lukewarm water spray feature of electronic toilets is to wash away contamination following defecation and to maintain hygiene. However, there have been few studies reporting the bacterial contamination in the spray water and in the gluteal and inguinal regions due to splashing following such spray washing. We have thus conducted a study to investigate the viable counts in the spray water and on the gluteal and inguinal regions following spray washing.

## Material and Methods

1. Viable count in spray water: Spray water was collected from ordinary houses and public toilets. Each sample of 0.1 mL was smeared on an individual culture medium, and cultured for 48 h under aerobic conditions at 37°C. The samples were then left for 6 days at room temperature, and colony numbers were counted. 2. Viable counts on the gluteal and inguinal regions following spray washing: The sample was collected from regions around 5 cm from the anus (gluteal region) and 10 cm anterior to the anus (inguinal region). The areas were cleaned with ethanol for disinfection and dried, and the sample was collected from the skin surface of the gluteal and inguinal regions using the XM-G agar medium for *E. coli* and coliform bacteria with the stamp method (control). Following spray washing, the sample was collected from the skin surface of the gluteal and inguinal regions using the same medium with the stamp method. The collected samples were cultured for 24 h under aerobic conditions at 37°C, and the colony numbers were counted.

## Results and Discussion

1. Viable count in spray water: The viable counts in the spray water of the ordinary houses, where toilets are used less frequently, were found to be 310.7±156.9 cfu/0.1 mL (*n*=80). In public toilets, which are more frequently used, the count was found to be 109.5±62.9 cfu/0.1 mL (*n*=28). These results show that regardless of the frequency of use, the spray water of toilets was contaminated by bacteria. Moreover, the viable counts in the spray water of the ordinary houses were higher than those of the public toilets. These results suggest that the spray water of the lukewarm water spray feature of electronic toilets, which had always been considered hygienic, was in fact contaminated by bacteria. It is conjected that this occurs due to the heating of mains water used for spraying, which volatilizes the chlorine in the spray tank and induces the growth of bacteria. 2. Viable counts on the gluteal and inguinal regions following spray washing: the *E. coli* and coliform viable count was 479±272 cfu (*n*=62) from the gluteal region and 16.9±9.9 cfu (*n*=16) from the inguinal region. These results suggest that the splashing from spray washing results in spreading of the *E. coli* and coliform bacteria in stool to the gluteal and inguinal regions.

Therefore, it is conjected to be possible that the use of water contaminated with bacteria at the time of spray washing, and also the spreading of *E. coli* and coliform bacteria after washing, increase infectiousness. Thus, it is necessary to understand the risk of bacterial infection, review hygiene management and look into measures for blocking pathways of bacterial transmission.

# Sub article: Molecular epidemiological analysis of methicillin-resistant Staphylococci in a neonatal intensive care unit

**Published:** 2012-05-23

**Authors:** Yasushi Takei, Kumi Yokoyama, Hideki Katano, Mami Tsukiji, Takayuki Ezaki, Seiki Tazume

**Affiliations:** 1 Department of Microbiology, Gifu University, Graduate School of Medicine, Gifu-shi Gifu, Japan; 2 Juntendo University School of Health Sciences and Nursing, Mishima-shi, Shizuoka, Japan; 3 Tokyo Healthcare University, Shinagawa, Tokyo, Japan; 4 Venex Research Institute, Atsugi-shi, Kanagawa, Japan; 5 Kanagawa Prefectural University of Human Service, Yokosuka-shi, Kanagawa, Japan; 6 Tokai University, Isehara-shi, Kanagawa, Japan

To investigate contamination and transmission of MRSA/MRCNS in the NICU of Hospital A (old NICU) and the relocated NICU (new NICU), we isolated and evaluated staphylococci from nurses’ palms, towels under the heads of neonates, infant incubators (including portholes and infant covers) and room air. Detection rates of MRSA/MRCNS isolated from different sample locations were 52.6% in the old NICU and 51% in the new NICU, which demonstrates that the nurses’ palms in both the old and new NICUs and indoor environment were contaminated with MRSA/MRCNS. In the old NICU, numerous MRSA and MRCNS strains (log 3.17±0.19 cfu/10 cm^2^) were identified in towels, and the implementation of improvement plans resulted in a decrease in the number of MRSA/MRCNS isolates (log 1.95±0.57 cfu/10 cm^2^) on the towels used in the new NICU. A homology study of MRSA/MRCNS strains by PFGE DNA restriction patterns identified genotypes that showed similar patterns in the nurses’ palms, towels, infant incubators and room air.

# Sub article: Isolation of toxigenic and nontoxigenic *Corynebacterium diphtheriae* strains upon preventive examination of hospital patients

**Published:** 2012-05-23

**Authors:** Irina Nicolaevna Aleseeva, Svetlana Evgenievna Ivanova, Natalia Vladimirovna Kasiyanenko, Elena Vladimirovna Lovschuk, Natalia Igorevna Dorogovtseva, Gennadiy Frankovich Rakitskiy

**Affiliations:** Territorial Mental Hospital, State Institution of Public Health, Khabarovsk

Mass immunization of children and adults with diphteria toxoid in Russia has resulted in a significant decrease in diphtheria incidence rate. As instructed by the Ministry of Public Health of Russia, epidemiological control over the diphtheritic infection has been routinely exercised in line with the infection prevention policy. For preventive purposes, the clinicodiagnostic laboratory of the Territorial Mental Hospital is engaged in bacteriological examination of all the patients hospitalized. The extent of examinations makes 800 people a month and 10,000 people a year. A significant portion of the inpatients belongs to an asocial group with decreased immunity and lacking vaccination against diphtheria.

The objective of the present research consisted in microbiological monitoring of circulation of toxigenic and non-toxigenic *Corynebacterium diphtheriae* strains.

Fauceal and nasal discharge was used as a material for examination. The material was inoculated onto dense Corynebacterium agar medium with 2% potassium tellurite solution added and incubated for 24–48 h at 37°C. Morphological, cultural and biochemical properties of isolated strains were examined. Toxigenic properties of *C. diphtheriae* were determined by a standard gel precipitation test. Biovariants and toxigenicity of the isolated strains were assessed. In recent years, we did not isolate any toxigenic *C. diphtheriae* strains. The rate of nontoxigenic strain isolation was low: 1–2 per 10,000 inpatients. However, from December 2010 to March 2011, we isolated 18 nontoxigenic strains of *C. diphtheriae* in 1,900 inpatients. Thus, the rate of isolation made 9.5 per 1,000 people examined—the figure agrees with the research data obtained at G.N. Gabrichevsky Research Institute of Epidemiology and Microbiology (Moscow).

According to their biochemical and cultural properties, 14 strains of the 18 *C. diphtheriae* strains isolated were ranked as *C. diphtheriae mitis* (78%) and 4 strains as *C. diphtheriae gravis* (22%). In the course of research, we found a difference in cultural properties between the strains isolated from our patients and the reference toxigenic *C. diphtheriae* strains obtained from L.A. Tarasevich Research Institute for Standardization of Control over Medical and Biological Specimens. According to the research carried out by G.N. Gabrichevsky Research Institute of Epidemiology and Microbiology (Moscow), 20.9% nontoxigenic *C. diphtheriae* strains, which were circulating in Russia in 1984–1998 and carrying a *tox*-gene, were isolated by PCR method. Results of the study evidence the potential role of the strains in generation of diphtheritic agent population. Moreover, there are strains of low toxigenicity level that may not be detected in routine research.

Thus, observations of *C. diphtheriae* strains isolated from patients during the preventive examination are an important component of epidemiological control over the diphtheritic infection. *C. diphtheriae mitis* was found to be prevailing (78%) among the isolated *C. Diphtheriae* strains. Diversity of cultural properties of the causative agent investigated requires a closer attention upon identification thereof. The strains isolated by the authors are subject to reidentification by PCR method to define if *tox-*gene carriage is likely. The data of microbiological monitoring should be used for prediction of epidemic and infectious development of diphtheritic infection processes.

# Sub article: Inhibition of biofilm formation and virulence factor expression via control of quorum sensing in pathogenic bacteria

**Published:** 2012-05-23

**Authors:** Tsukasa Ikeda, Tomohiro Morohoshi

**Affiliations:** Department of Material and Environmental Chemistry, Graduate School of Engineering, Utsunomiya University, Yoto, Utsunomiya, Japan

Quorum sensing is a well-known bacterial communication mechanism and several kinds of *N*-acyl-l-homoserine lactones (AHLs, Fig. 1) have been identified as one of the quorum sensing signal compounds in gram-negative bacteria (1). As a lot of bacteria species control the expression of genes responsible for bioluminescence, secretion of virulence factors, expression of pathogenicities or forming biofilm via quorum sensing, the control methods of quorum sensing have been extensively studied. Herein, report about several results about inhibition of quorum sensing in gram-negative bacteria.

**Fig. 1:** Structure of AHLs.

Several kinds of AHL degrading enzymes have been isolated, and the use of them for inhibition of quorum sensing has been studied and showed good effects. Some kinds of synthetic AHL analogues can act as inhibitors of quorum sensing. Especially, *N*-acyl-cyclopentylamines (Cn-CPAs) showed inhibitory effects on quorum sensing including bioflm formation in *Psuedomonas aeruginosa*, *Serratia marcescens* and several kinds of gram-negative bacteria without effects on bacterial cell growth (2). Cyclodextrins (CDs), a series of oligosaccharide, can make a complex with AHLs in a bacterial culture medium, and quorum sensing in *P. aeruginosa* and *S*. marcescens was inhibitted by adding CDs to a bacterial culture medium (3). For the development of this method, several kinds of modified CDs and CD-immobilized gel sheets were synthesized and showed strong inhibitory effects on quorum sensing.

These quorum sensing inhibition methods may one of the useful methods to inhibit biofilm formation and bacterial infectious disease without antibiotic-resistant bacterial problem.

## References

[1] Greenberg EP (1997). Quorum sensing in gram-negative bacteria. ASM News.

[2] Morohoshi T, Shiono T, Takidouchi K, Kato M, Kato N, Kato J (2007). Inhibition of quorum sensing in Serratia marcescens AS-1 by synthetic analogs of N-acylhomoserine lactone. Appl Environ Microbiol.

[3] Ikeda T, Inoue Y, Suehiro A, Ikeshoji H, Ishida T, Takiguchi N (2002). The effects of cyclodextrins on autoinducer activities of quorum sensing in Pseudomonas aeruginosa. Macro Chem.

# Sub article: Deletion of alarmon synthetase altered physiology and biofilm formation of *Bordetella pertussis*

**Published:** 2012-05-23

**Authors:** Kentaro Sugisaki, Tomoko Hanawa, Hideo Yonezawa, Takako Osaki, Satoshi Kurata, H. Kawakami, Shigeru Kamiya

**Affiliations:** 1 Department of Infectious Diseases School of Medicine, Kyorin University, Tokyo, Japan; 2 Department of Anatomy, School of Medicine, Kyorin University, Tokyo, Japan

The ability of the cell to adapt to nutrient limitation depends on its capacity to change its gene expression immediately when nutrients are depleted. One important mechanism for regulating this change in gene expression is the stringent response. The guanosine tetraphosphate (ppGpp) molecule, which is an effector of the stringent response, binds to RNA polymerase and regulates transcription of various genes. During infection in host, bacteria need to adjust rapidly to the environments such as amino acid starvation and/or other nutritional starvation. Stringent response in *Bordetella pertussis* has not been examined so far. To elucidate the role of stringent response, we constructed the deletion mutants of ppGpp synthetase and assessed the biofilm formation. According to the genome sequences of *B. pertussis*, *relA* and *spoT* were identified as the genes encoding ppGpp synthetase. Therefore, *relA* and *spoT* double mutant of *B. pertussis* was constructed and characterized.

Double mutant designated with *relAspoT* double mutant strain grew similarly to wild type strain at 35°C in the Stainer-Scholte culture medium. When the concentration of glutamic acid in the medium was decreased, *relAspoT* double mutant strain lost the negative regulation of growth occurring by nutritional starvation. Furthermore, ability of biofilm formation was significantly decreased by *relAspoT* double mutant. Scanning electron micrographs of bacterial cells on the glass showed few extracellular structures of *relAspoT* double mutant strain.

As a result, it is suggested that ppGpp regulates growth rate under nutritional starvation, cell morphology and biofilm formation in *B. pertussis*.

# Sub article: Inhibition of *Staphylococcus aureus* biofilm formation and nasal colonization by a commensal bacterium *Staphylococcus epidermidis*

**Published:** 2012-05-23

**Authors:** Tadayuki Iwase, Yoshio Uehara, Hitomi Shinji, Akiko Tajima, Hiromi Seo, Sinya Sugimoto, Toshihiko Agata, Koji Takada, Yoshimitsu Mizunoe

**Affiliations:** 1 Department of Bacteriology, The Jikei University School of Medicine, Tokyo, Japan; 2 Department of General Medicine, Kochi Medical School, Nankoku, Japan; 3 Department of Environmental Health, The Jikei University School of Medicine, Tokyo, Japan; 4 Department of Biochemistry, The Jikei University School of Medicine, Tokyo, Japan

Commensal bacteria are known to inhibit pathogenic colonization; however, complex host–microbe and microbe–microbe interactions have made it difficult to gain a detailed understanding of the mechanisms underlying the inhibition of colonization. In our study, by using a collaborating approach of bacteriology and epidemiology, we show that the serine protease Esp secreted by a subset of *Staphylococcus epidermidis* inhibits *Staphylococcus aureus* biofilm formation and nasal colonization.

Our findings are as follows:

1) A subset of *S. epidermidis* produced Esp, which inhibited biofilm formation and destroyed the preexisting biofilms formed by *S. aureus*, including those by multidrug resistant strains. 2) Epidemiological studies indicated that the presence of Esp-positive *S. epidermidis* in the nasal cavities of human volunteers correlates with the absence of *S. aureus*. 3) Esp enhanced the susceptibility of *S. aureus* in biofilms to human beta-difencin2 (hBD2), an antimicrobial peptide secreted by keratinocytes. Our recent data indicate that Esp also enhanced the susceptibility of multi-drug resistant strains in biofilms to hBD2. 4) *In vivo* studies have shown that purified Esp and Esp-positive *S. epidermidis* eliminated *S. aureus* nasal colonization.

These findings imply that Esp hinders *S. aureus* colonization *in vivo* through a novel mechanism of bacterial interference; this knowledge could lead to the development of novel approaches for the prevention and treatment of *S. aureus* infections, including infections caused by multiple drug-resistant strains. Studies at the strain level and interdisciplinary approaches will further enhance our understanding of microbial ecology, including host–microbe interactions and the relationships between commensal bacteria and infectious pathogens.

# Sub article: Probiotics and innate immunity: regulation of anticancer activity

**Published:** 2012-05-23

**Authors:** Vyacheslav Abramov, Valentin Khlebnikov, Irina Chikileva, Vadim Sakulin, Igor Kosarev, Raisa Vasilenko, Mikhail Kiselevsky, Vyacheslav Melnikov

**Affiliations:** 1 The Institute of Immunological Engineering, Lyubuchany, Moscow Reg., Russia; 2 Russian Oncological Centre, RAMS, Moscow, Russia; 3 International Science and Technology Center, Moscow, Russia

Probiotics improve innate anticancer defense capacity; however, the mechanisms of this activity are sill little studied. We gathered a collection of *Lactobacillus* and *Bifidobacterium* strains isolated from various regions of Russia and Armenia. Lactobacilli were identified using API 50 CHL techniques and sequencing of 16S gene RNA. Identification of bifidobacteria was performed by PCR. The strains effectively growing on nutrient medium and positively tested for their stability to gastric and intestinal stresses were selected for immunological studies. Bifidobacteria were additionally tested for their stability to oxygen. *L. casei* DN 114001 (Danone, Actimel) and *B. animalis lactis* DN 173001 (Danone, Activia) were used as controls. All the studied strains of lactobacilli and bifidobacteria did not induce the production of IL-4, key mediator of allergic reactions, in monocytes. At the same time, a stimulation of the synthesis of IL-6 responsible for B lymphocyte maturation and humoral immunity development was registered. These data indicated probiotic participation in regulation of Th1/Th2 balance and their preventive role in allergic disease appearances. Bifidobacterium strains under study inhibited IL-12 synthesis in immature dendritic cells (DC_S_) and stimulated IL-10 production blocking DC_S_ maturation. The studied *L. casei* DN 114001, *L. plantarum* 191 strains displayed a stimulatory effect on the immune cells (NK, monocytes/macrophages and DCs) and antitumor effect on human erythroblast leukemia cells K562. Several studies established a key role of tumor cell–platelet interaction as one of the earliest processes favoring tumor metastasis. Therefore, we aimed to determine the effect of lactobacilli and their culture liquid (CL) on the adhesion of highly metastatic breast cancer cell line MCF-7 to a platelet-coated surface under static conditions *in vitro*. CL of *L. casei* DN 114001, *L. plantarum* 191 and *L. plantarum* 189 strains, as well as live bacteria, caused prominent inhibitory effect on the binding of tumor cells and platelets, which exceeded the effect of the fucoidan LS (positive control). Similar results were obtained in the tests with another tumor cell line DU-145 (human prostate cancer). The antimetastatic effect of CL of *L. casei* DN 114001 and *L. plantarum* 191 strains was tested *in vivo* using Balb/c-nude mice. Tumor cells were injected subcutaneously into animals to provoke melanoma B-16 in them. On the 28th day, this melanoma was removed surgically. On the 56th day, CL immunorestorative effect was observed in mice. After surgical removal of the tumor, intensive formation of metastases was observed in the lungs and pleura of the animals, which had not received CL. As a result, 100% of the animals from the control group had metastases. The number of the metastases varied from 8 to 15. The most mice that received CL had no metastases. These studies allowed one to ascertain for the first time CL antimetastatic effect of lactobacilli in conditions of tumor surgical removal. Isolation of surface factors of *L. casei* DN 114001 and *L. plantarum* 191 inhibited the interaction of the tumor cell with thrombocytes, and determination of the structure of these factors would enable to create new medicinal preparations for a complex therapy of oncological patients in postoperative period. A search for lactobacillus strains with upregulated activity to activate NK cells in a combination with effective metastasis inhibition and development on their base of functional nutrition products is a new and perspective direction in prophylaxis and treatment of cancer diseases.

# Sub article: Possible health promoting benefits of heat-killed *Lactobacillus gasseri* TMC0356, a new selected immune regulatory probiotics strain

**Published:** 2012-05-23

**Authors:** Kenji Miyazawa, Fang He, Kazutoyo Yoda, Manabu Kawase, Akira Kubota, Masaru Hiramatsu

**Affiliations:** Technical Research Laboratory, Takanashi Milk Products Co., Ltd, Yokohama, Japan

*Lactobacillus gasseri* TMC0356 (TMC0356) was a human original probiotic strain found in 1997. This bacterium can activate macrophage to secret both inflammatory and anti-inflammatory cytokines and induce IgA production from Peyer's patches apparently. Animal and human clinical studies found that TMC0356 can significantly improve allergic symptoms and the related immunity, protect host animal against virus infection and suppress the tumors growth (1).

Recently, emerging scientific evidence has revealed that inactive probiotic cells may confer apparent health benefits on host animals. Compared with living cells, inactive cells are easier and more convenient to handle and can be used in different food product lines in addition to fermented milk or yoghurt, which are popular probiotic foods.

In the present study, the heat-killed TMC0356 induced IL-12 production from macrophage-like cell line J774.1, inhibited the growth of transplanted tumors in mice and protected mice from influenza virus infection. Furthermore, the heat-killed TMC0356 also expressed the promoting anti-obesity effects and potent possibility to alter age-related immunosenescence in host animals.

These results suggested that heat-killed TMC0356 can contribute to the health of host animal as well as its living cells. Heat treatment could be considered as one of the effective and practical ways to expand the application of probiotics in various foods and nutrition supplements.

## References

[1] Miyazawa K, He F, Kawase M, Kubota A, Yoda K, Hiramatsu M (2011). Enhancement of immunoregulatory effects of *Lactobacillus gasseri* TMC0356 by heat treatment and culture medium. Lett Appl Microbiol.

# Sub article: Effects of probiotics on gut microbiota composition in familiar Mediterranean fever and Crohn's disease patients

**Published:** 2012-05-23

**Authors:** Anahit M. Manvelyan, Marine A. Balayan, Elya S. Pepoyan, Susanna S. Mirzabekyan, Lena M. Malkhasyan, Vardan V. Tsaturyan, Astghik Z. Pepoyan

**Affiliations:** 1 Food Security and Biotechnology Department, Armenian State Agrarian University, Teryan, Yerevan, Republic of Armenia; 2 Yerevan State Medical University after M. Heratsi, Republic of Armenia

## Background

The incidence of patients with Crohn's disease (CD) and with familiar Mediterranean fever (FMF) disease has been increasing dramatically over the last few years in world. FMF is an inherited disorder that usually occurs in people of Mediterranean origin—including Sephardic Jews, Arabs, Armenians and Turks, but it may affect any ethnic group. The frequency of FMF patients in the Armenian population is 0.25%, unusually high. The ratio of obligate heterozygotes to healthy persons is 0.14. While there is no cure for this disorder, one may be able to relieve signs and symptoms of FMF—or even prevent them altogether—by adhering to a highly effective treatment: colchicine, 1–2 mg/day, for life. The drug's mode of action is unknown, but this molecule is able to inhibit attacks or prolong the intervals between them. It may also prevent or delay the appearance of renal complications in 2/3 of the patients. While colchicine is effective in treating FMF, it is not without side effects: many patients taking colchicines complain of general gastrointestinal upset.

## Objectives

The aim of these investigations was characterization and comparative analysis of gut bacterial diversities of FMF and CD patients with or without probiotics treatment.

## Methods

110 FMF (with or without colchicine treatment) and 20 CD volunteer patients were enrolled in our study at different hospitals in Yerevan, Armenia. The patients’ fecal bacterial diversities were investigated using culture-based method and 16S rDNA libraries construction.

## Results

The results of our investigations showed that there is an obvious deviation in gut bacterial diversities of Armenian FMF and CD patients. Due to few numbers of patients with probiotic treatment, we did not found any association between gut bacterial composition and probiotic use of patients. Taking into account the results of investigations, we hypothesize that the corrective probiotics therapy may be recommended for the patients only based on their gut microbiota composition. This work was supported by ISTC A-732 and A-1227.

# Sub article: Development of probiotic preparations and functional food products on the base of lactic acid bacteria

**Published:** 2012-05-23

**Authors:** Nadiia K. Kovalenko

**Affiliations:** Institute of Microbiology and Virology, National Academy of Sciences of Ukraine, Kiev, MSP, Ukraine

New modern approach for probiotic strain selection has been described. Distribution of lactic acid bacteria (LAB) in nature have been demonstrated on the basis of experimental results. It has been revealed that optimal characteristics of LAB depend on their environment. New interpretation of LAB ecology has been presented.

The specificity of influence of environmental conditions of natural substrates is the subject of scientific interest in term of study taxonomy of LAB and their biological properties. The collection of LAB strains isolated from different ecological niches has been created. The identification of these strains has been conducted by the proposed scheme of successive steps of differentiation using both phenotypic and genetic analysis (PCR, RAPD PCR, RFIP and 16S rDNA sequencing).

It has been determined that selected strains possess probiotic activity, as well as manifest antiviral activity and the adaptive ability. The regulation of their activity in some ecosystems has been developed. Probiotics for protection human and animal organisms against acute gastrointestinal diseases have been manufactured. Among them are Lactosan, Bovilact, Streptosan, Litosil, Lactin and Gerosan. The functional food products based on probiotic cultures for elderly people have been developed (Gerolact and Lactogerovit).

The results obtained allow to produce probiotics and functional food products with new comprehensive method, considering modern requirements as for bacteria identification.

# Sub article: Beneficial effects of fermented milk containing *Lactobacillus* GG on DSS-induced colitis in mice

**Published:** 2012-05-23

**Authors:** Kazutoyo Yoda, Fang He, Kenji Miyazawa, Manabu Kawase, Akira Kubota, Masaru Hiramatsu, Fang Yan

**Affiliations:** 1 Technical Research Laboratory, Takanashi Milk Products Co., Ltd., Yokohama, Japan; 2 Department of Pediatrics, Vanderbilt University School of Medicine, Nashville, TN, USA

Probiotic bacteria are microorganisms that benefit the host by preventing and/or treating diseases. *Lactobacillus* GG (LGG), a well-known probiotic bacterium, secrets two functional proteins, P40 and P75, which suppress intestinal inflammation. The purpose of this study is to detect these functional proteins from a commercial fermented milk product containing LGG (LGG-milk) and investigate actions of LGG-milk on cultured epithelial cells and DSS-induced colitis in mice.

P40 and P75 were detected from supernatant of LGG-milk by Western-blotting analysis using anti-P40 and anti-P75 antibodies.

Young adult mouse colon (YAMC) cells were cultured with 1:100 to 1:2,000 diluted supernatant of LGG-milk for 2 h. Cellular lysates were prepared for Western-blotting analysis using anti-EGF receptor-Tyr1068 and anti-phosphorylated Akt antibodies to detect EGF receptor and Akt activation, respectively. LGG-milk activated EGF receptor and Akt in a concentration-dependent manner in the YAMC cells.

To evaluate the preventive effect of LGG-milk on DSS-induced colon epithelial injury and colitis, female C57BL/6 mice were treated with 3% DSS in drinking water for 4 days to induce colon injury and acute colitis. 500 µl of LGG-milk was gavaged to mice 6 days before and during DSS treatment. Inflammation and injury was assessed using a score system. DSS-induced colitis (score: 6.2±0.84) was significantly decreased by LGG milk treatment (score: 3.4±3.14). The shortening of the colon induced by DSS (6.38±0.39 cm), as a marker for colitis, was prevented by LGG-milk treatment (7.48±0.48 cm). These results indicate that LGG-milk exerts a preventive effect on DSS-induced colitis in mice.

# Sub article: Influence of probiotic Enterococci on the gastrointestinal tract of rats before and after the treatment of antibiotic-associated dysbiosis

**Published:** 2012-05-23

**Authors:** Alexander Suvorov, Ludmila Gromova, Yuri Borschev, Alena Karaseva, Andrei Gruzdkov

**Affiliations:** 1 Institute of Experimental Medicine RAMS, St. Petersburg, Russia; 2 Pavlov Institute of Physiology RAS, St. Petersburg, Russia

Implementation of probiotics containing lactic acid bacteria (LAB) have been proved to be useful in many clinical conditions including antibiotic associated diarrhea. However, little is known about the specialties of their colonization in mammals and effects on the digestive system when LABs are used to prevent or treat the intestinal dysbiosis. The purpose of this study was to investigate the influence of probiotic Enterococci on the condition of the experimental animals, their microbiota and the activity of key digestive enzymes. Male Wistar rats were getting milk fermented products containing *E. faecium* L5 (ermB-labeled derivative of probiotic strain L3). Bacteria were introduced into the stomach of the healthy animals or the rats treated with antibiotics (ampicillin and metronidazole, 3 days). At the end of the experiment, samples of chyme and epithelium were taken from different parts of the gastrointestinal tract and studied by bacteriological and biochemical methods. It was shown that the administration of probiotic did not affect on the rat's condition and behavior. Dysbiosis associated with the intake of antibiotics was confirmed by the changes in microbiota and by the appearance of dyspeptic symptoms. Pathological symptoms disappeared after the administration of Enterococci and the original intestinal microbiota was restored.

Usage of probiotic led for colonization of jejunum, ileum and all parts of large bowel with *E. faecium* L5. Moreover, erythromycin-resistant Enterococci were detected in the stomach of some animals. No bacteria were found in the spleen, liver, heart or the blood of rats. It was typical for all rats treated with probiotic: the reduction of maltase activity of the epithelium and the increasing of activity of alkaline phosphatase in the chyme of the small intestine. Rats after the consumption of antibiotics had lower values of maltase activity in the chyme of large bowel in contrast to healthy rats or animals with dysbiosis receiving probiotic therapy.

Strain *E. faecium* L5 was able to temporarily colonize the gastrointestinal tract of the animals and caused the disappearance of dysbiosis and changes in the activity of intestinal enzymes (in particular maltase activity in the cavity of the colon). The specific effects provided by strain *E. faecium* L5 (L3) on the activity of maltase and alkaline phosphatase should be considered when comparing this strain with other probiotic microorganisms.

# Sub article: Development of the Caucasian lactic acid bacterial culture collection for the potential industrial use

**Published:** 2012-05-23

**Authors:** Nina Chanishvili

**Affiliations:** Eliava Institute of Bacteriophage, Microbiology and Virology, Tbilisi, Georgia

The traditional Caucasian dairy products (such as Matsoni—the Caucasian yogurt like product, cheeses, etc.) remain almost unexplored. The commercial and, especially, health beneficial value of these products are poorly studied. At the same time, e.g., Matsoni is historically known as a supplement of the infant's diet, remarkable remedy against intestinal disorders, burned wounds and dermatitis, in domestic cosmetics for improvement of skin and hair conditions, also as a base of special food-preserving solutions. Hence, it can be considered as a potential pool of the strains with antimicrobial activity against pathogens causing human and animal diseases, as well as food-spoilage micro-organisms.

Thus, the bacterial cultures isolated from the home made dairy products may be considered as a potential pool of genes determining antibacterial activities against human and animal pathogens as well as food taste and quality spoilage microorganisms.

Traditional methods including chromatography (HPLC analysis of fermentation and products), API tests (bio Merieoux) along with modern genetic techniques for identification of strains, such as PCR and DNA sequencing of 16S-23SrRNA intergenic regions have been applied for strain identification.

During the past years, about 50 homemade Matsoni samples and 20 cheese samples have been collected in different parts of Georgia. Approximately 400 lactic acid bacterial cultures have been isolated. The number of components in the homemade Matson samples were 2 at minimum and 7 at maximum, and diversity of lactic acid strains remarkably varied in accordance with geographical origin of the starter. In particular, presence of the following bacterial and yeast species have been determined: *Lactobacillus delbrueckii* ssp. *bulgaricus*, *Lactobacillus delbruekii* ssp. *lactis*, *Lactobacillus casei*, *Lactobacillus acidophilus*, *Streptococcus thermophilus*, *Lactococcus cremoris*, *Lactococcus lactis*, *Enterococcus* sp., *Saccharomycetes cerevisiae*, *Candida lucitaniae* [5, 6, 7]. It was demonstrated that only *L. delbruekii* ssp. *bugaricus* and *S. thermophilus* are responsible for clot formation and taste; however, the taste of Matsoni is different from yogurt. Another bacterial species, *E. durans* is also frequently found in the Matsoni samples, which is an essential part of the Italian cheese Mozzarella. According to the present knowledge, *Enterococci* do not play significant role in Matsoni, but some of them are having distinguished antibacterial properties.

A basic culture collection of the endemic dairy strains has been developed. Only 10–20% of the strains from this culture collection is fully or sometimes partially characterized by now. It appeared that about 30% of the tested lactic acid bacteria are antagonistic to the various pathogens, while only 20% out of them showed the best biotechnology potential. The studies need to be continued for selection of the most efficient probiotics and the strains with the strong proteolytic activities.

# Sub article: Probiotic ‘Lactovit-K’ fights against coccidiosis of poultry and honey bee diseases

**Published:** 2012-05-23

**Authors:** Nina N. Gavrilova, Irina A. Ratnikova, Vyacheslav G. Melnikov

**Affiliations:** 1 Institute of Microbiology and Virology, Almaty, Kazakhstan; 2 International Science and Technology Center, Moscow, Russia

The coccidiosis of poultry is sometimes difficult to treat because of the associative character of the disease—coccidium aftereffects with enteropathogenic bacteria infection. It is not always taken into account. Secondary infection makes the primary disease much more complicated. Septic form of colibacteriosis often becomes a cause of the animal death. In order to fight against this secondary bacterial superinfection, we performed a probiotic preparation ‘Lactovit-K’, which contains lactobacilli, propionibacteria and vitamins B_3,_ B_8_, B_12_. The probiotic appeared to be capable of inhibiting growth of pathogenic *E. coli* and *Salmonella in vitro*.

Experiment was carried out using the livestock of 8,000 chickens with coccidiosis. The preparation was given to the chickens with forage during 10 days. In the end of treatment period, the general state of the chickens improved, and there was almost no loss of the birds. The viscera had no any pathological changes; mucous membrane of the bowel was normal. Pathogenic *E. coli* were not observed. In only some chickens, moderate amount of coccidium oocysts (3–5 ones in the microscopic field) was found. Chickens in the control group did not grow well; they had diarrhea, and their feathers were disheveled and dirty. Considerable loss of the chickens was seen. Laboratory examination of dead birds revealed inflammation of bowel mucous membrane, heavy coccidial and pathogenic *E. coli* infections. So, the research results have proved the possibility of the use of probiotics against coccidiosis.

Lactovit-K has showed its high effectiveness against honey bee diseases too. When using the preparation in combination with carbohydrate and protein feed, we observed the reduction of incidence of American foulbrood (bacterial disease of bees) by 97% and of *Varroa jacobsoni* (tick-born disease) by 74%. In combination with sugar syrup, the effectiveness of Lactovit-K regarding American foulbrood was 80%, and the incidence of *V. jacobsoni* was reduced by 70%.

# Sub article: Biological active compounds from probiotic bacteria

**Published:** 2012-05-23

**Authors:** Galina Novik, Elena Kiseleva, Vyacheslav Melnikov, Andrzej Gamian, Yuriy Knirel, Estera Szwajcer Dey

**Affiliations:** 1 Institute of Microbiology BNAS, Minsk, Belarus; 2 Institute of bioorganic chemistry BNAS, Minsk, Belarus; 3 International Science and Technology Center, Moscow, Russia; 4 Institute of Immunology and Experimental Therapy, PAS, Wroclaw, Poland; 5 N.D. Zelinsky Institute of Organic Chemistry, RAS, Moscow, Russia; 6 Lund University, Pure and Applied Biochemistry, Lund, Sweden

Probiotic bacteria are natural inhabitants of human gastrointestinal tract. Extensive investigations of biological active compounds from probiotic bacteria are aimed primarily at the development of highly effective therapeutic products. Among these substances, there are bacterial polysaccharides and polar lipids. Therefore, it is important to identify the biologically active compounds and to determine their structures. Chemical, NMR spectroscopic and serological studies revealed a significant structural heterogeneity of the polysaccharides, which in turn might influence their biological activity, namely the antigenicity. A polysaccharide of α1,4-glucan type was isolated from *Bifidobacterium adolescentis* 94-BIM. The major polysaccharide specific for strain *B. bifidum* BIM B-465 was found to be a branched glucogalactan with a heptasaccharide repeating unit. *B. longum* BIM B-476D produced several polysaccharides, including a glucosylated ribitol teichoic acid and a polymer with a branched pentasaccharide 1-phosphate repeating unit. Current research on biological active compounds from probiotic bacteria is aimed primarily at the elaboration of glycoconjugates products. By employing a new modification of supercritical fluid of carbon dioxide (scCO_2_), polar lipids of lactic acid bacteria and bifidobacteria can be very effectively extracted. TLC analysis of scCO_2_ fractions showed predominance of glycolipids and phospholipids. Glucose and galactose were detected as sugar components of isolated glycolipids. Glycolipids with high immunoreactivity were identified by enzyme linked immunosorbent assay (ELISA). Additionally we received data suggesting a possible role of probiotic bacteria of genera *Bifidobacterium* and *Lactobacillus* in pathogenesis of autoimmune thyroid disease (ATD). Using proteins and immunogenic peptides databanks and relevant computer programs, homology was established between the amino acid sequences of thyroid peroxidase (TPO) and thyroglobulin (Tg), which are potential B- and T-cell epitopes of these autoantigens and of a number of proteins of *Bifidobacterium* and *Lactobacillus*. We also have found nonprotein components of cells of *Bifidobacterium bifidum* 791, *B. adolescentis* 94 BIM, *B. longum* B379M and *Lactobacillus plantarum* B-01 that cross-react with human serum autoantibodies (AAbs) to TPO and Tg and compete with relevant antigens for the binding of these AAbs in ELISA. Moreover, a three-fold difference was disclosed between the probability of detection of Abs to the antigens of *L. plantarum* B-01 and *B. bifidum* 791 in serum samples containing and not containing AAbs to TPO, which are serologic markers of ATD. On the whole, our data are arguments in favor of the assumption of a possible role of PM of genera *Bifidobacterium* and *Lactobacillus* in triggering ATD through the mechanism of molecular mimicry. In prospect, the scCO_2_ isolation technology of probiotic glycoconjugates may be combined with metabolic engineering and immunological studies in technologies aimed at manufacturing highly effective therapeutic products.

# Sub article: The role of probiotics in rehabilitation of sportsmen after postmatch exertion

**Published:** 2012-05-23

**Authors:** Gagik Hoveyan, Marine Badalyan, Ani Hoveyan, Elya Pepoyan, Vardan Tsaturyan, Astghik Pepoyan

**Affiliations:** 1 Buniatian Institute of Biochemistry, National Academy of Sciences, Republic of Armenia; 2 Yerevan State Medical University after M. Heratsi, Republic of Armenia; 3 Food Security and Biotechnology Department, Armenian State Agrarian University, Republic of Armenia

One of the most important problems of modern biomedical science and clinical medicine is the revealing of the bimolecular mechanisms of wide scope of physical and emotional exertion and rehabilitation of sportsmen by administration of probiotics, prebiotics and food supplements.

It is known that during and after sport fatigue, the inhibition of organism adaptation takes place. That is the universal biological process, by means of which the organism can get accustomed to the various changes of the external and internal mediums. In the process of adaptation, the time-depending organization of organism undergoes changes as well different nervous, hormonal and immunologic displacements occur bearing wave-like character. In the each state of adaptation, the homeostatic factors supporting the adaptive properties occurred in previous stages simultaneously neutralize the negative damaging properties of them. Adaptation needs coordination of regulation and integration of endocrine, nervous and immune systems, which regulate processes of metabolism, generation of energy, increase of antiradical defense and redistribution of substances in tissues of organism.

On the other hand, probiotics are considered as important factors not only for the restoration of commensal microbiota but also for increasing adaptation–compensatory possibilities of organisms. Probiotics possess antifree radical, immune stimulating activities and possibility to take part in processing, distribution and absorption of nutrients in intestine.

The aim of these investigations was the characterization and comparative analysis of gut bacterial diversities of sportsmen-groups. Thirty-five sportsmen were involved in our investigations. The fecal samples from sportsmen were gathered a week before and after the championships, and the fecal bacterial diversities by classical culture-based method were investigated.

The results of our preliminary investigations showed that there is a deviation in gut main bacterial groups depending on championship period. The role of probiotics as important agents of rehabilitation of sportsmen at physical exertion and overtraining is also discussed.

# Sub article: Breakdown of *Bifidobacterium* flora by species in the process of intestinal biocenosis development in newborns and in dysbiotic individuals

**Published:** 2012-05-23

**Authors:** Elena Fedorovna Zavgorodnyaya

**Affiliations:** Khabarovsk Research Institute of Epidemiology and Microbiology, Federal State Institution of Science under the Federal Service for Supervision in the Field of Consumer Right Protection and Human Welfare, Russian Federation

Study was made of *Bifidobacteria* species, adhesive and antagonistic activity thereof in 25 pregnant women and their newborns (in the process of microflora acquisition from the 1st through the 30th day of life)—180 tests, as well as in dysbiotic cases of children over 3 years of age and adults—257 tests.

Bifidobacteria are isolated from the gut culture of 64.0% of children since the first day of life in quantity of 3.3 lg/g; by the 5th day of life, the quantity thereof increases up to 8.3 lg/g. However, by the age of one month, *Bifidobacterium* quantity amounts to 9.0 lg/g in only 52.2% of children. Stable prevalence of *Bifidobacteria* in the biocenosis of infants aged up to 10 days of life was only registered in 20% of children, of infants aged 10 through 15 days—in 44.0% of children (it is these figures that are registered in the majority of newborns in European Russia); 24.0% of newborns demonstrated delay in bifidoflora colonization up to as late as the 20th–30th day of life, while 12.0% of infants were not dominated by *Bifidobacteria* even by the end of the first month of life, i.e., the fact of *delay in bifidoflora development* was established. Most likely, this was due to the transfer of *Bifidobacteria* of lower biological activity from the mother to the newborn, which results from the prevalence of dysbacteriosis in people, including pregnant women.

Intestinal microflora of pregnant women presented with dominance of *B. adolescentis* and *B. breve* (total of 86.4%). Newborns aged 1 through 5 days were also dominated by *B. adolescentis* (84.0%) and *B. breve* (16.0%). Thus, a second *specific feature of bifidoflora development* in newborns was identified, namely: child-specific term of an initial increase (from the first to the fifth day of life) and a subsequent decrease (more frequently on the fifth–fifteenth day) in both the total number of *Bifidobacteria* and their share in the intestinal microflora (45.4% and 63.6%, respectively).

It has been found out that, in the process of the decrease in the quantity of *Bifidobacteria* species, rearrangement thereof takes place, and in 80% of cases, *B. adolescentis* and *B. breve* are replaced by the so-called ‘suckling’ and other (*B. infantis*, *B. lactensis* and *B. bifidum*) species or associations thereof, which dominate in the biocenosis of healthy infants during the entire period of breast-feeding.

Species rearrangement of *Bifidobacteria* is preceded by a decrease in antagonistic and adhesive activity thereof, which was registered in 42.8% of *Bifidobacterium* strains isolated from infants aged 5–15 days. Apparently, species rearrangement of bifidoflora makeup occurs in stages and may be described as follows: *first stage*—decrease in a physiological activity of the prevalent species of *Bifidobacteria* (e.g., *B. adolescentis*); *second stage*—decrease in the number of *Bifidobacteria* of said species; *third stage*—increase in the number of *Bifidobacteria* of other species; (e.g., *B. lactentis*) with a gradual domination thereof in the biocenosis. The same consistent pattern is also seen in intestinal dysbacteriosis development, but the succeeding *Bifidobacteria* species prove to be other than the above. Thus, *B. adolescentis* dominates in the large intestine of 67.7% healthy adults and children over 3 years old in all seasons, both in monocultures and in associations with other species. In dysbacteriosis cases, the same species remains prevalent but the quantity of genus bifidum proves significantly increased (14.3% in eubiotic condition and 25.6% in dysbacteriotic condition). That is, *rearrangement of Bifidoflora makeup* takes place in the course of development of intestinal dysbacteriosis.

From 81.8% of dysbacteriotic individuals, *Bifidobacteria* of lower antagonistic activity (in normocenosis—from 43.3% only) and lower adhesive activity (46.7% vs 78.2%) were most frequently isolated.

Thus, the process of rearrangement of makeup during bifidoflora development and dysbacteriosis development follows the same biological mechanism.

# Sub article: Efficacy of the probiotic strain *Clostridium butyricum* MIYAIRI 588 on poultry and piglet zootechnical performance and prevention of necrotic enteritis

**Published:** 2012-05-23

**Authors:** Motomichi Takahashi, Miroslava Piskoríková, Kaoruko Yuge, Kentaro Oka, Koji Uno, Elinor McCartney, Shigeru Kamiya

**Affiliations:** 1 Miyarisan Pharmaceutical, Japan; 2 Department of Infectious Diseases, Kyorin University School of Medicine, Japan; 3 EU Pen & Tec Consulting, Spain

The 2006 EU ban on antibiotic growth promoters has stimulated research on alternative products to improve zootechnical performance and reduce problems such as necrotic enteritis (NE). *Clostridium butyricum* MIYAIRI 588 (CBM 588) is a viable spore probiotic strain, approved for human pharmaceutical products in Japan and Asia and as a feed additive in EU. From 2006 to 2010, we carried out four large field studies with broilers and five studies with weaned piglets in several EU countries.

Zootechnical data from all studies were analyzed, including daily weight gain, feed intake, feed efficiency (feed:gain) and mortality. Data were then tested for homogeneity and pooled for meta-analysis, where *p*≤0.05 was considered significant, and 0.05 <*p*≤0.10 a near-significant trend. We also tested the ability of CBM 588 to reduce NE lesions in a pilot study in broilers that modeled naturally occurring NE.

The meta-analysis of four broiler field trials (1–42 days of age) demonstrated a significant (2.3%) improvement in feed:gain (1.74 versus 1.70 for control and CBM 588 broilers, respectively, *p=*0.0001) and a 1.8% decrease in feed intake (95.7 versus 94.0 g/day for control and CBM 588 broilers, respectively, *p=*0.0163). No significant differences were detected in mortality.

In the pilot broiler NE study (0–35 days of age), there were no significant differences in performance (growth, liveweight, feed intake, feed efficiency, mortality and European Production Efficiency Factor) but a clear dose-response at 32 days of age related to reduction of the severity of NE lesions (*P*<0.05).

The meta-analysis of five piglet field studies demonstrated a significant (4.2%) improvement in feed:gain (1.60 versus 1.67 for CBM 588 and control piglets, respectively, *p*=0.0019) and a 4.8% increase in mean daily gain (380.55 versus 363.22 g/day for CBM 588 and control piglets, respectively, *p*=0.0048). No significant differences were detected in feed intake (602.97 versus 602.03 g/day for CBM 588 and control piglets, respectively, *p=*0.9222).

The meta-analysis data indicate that CBM 588 is an effective zootechnical feed additive in broilers and piglets and may help reduce NE.

# Sub article: Reactivation of latent HIV-1 by a wide variety of butyric acid-producing bacteria

**Published:** 2012-05-23

**Authors:** Kenichi Imai, Kiyoshi Yamada, Muneaki Tamura, Kuniyasu Ochiai

**Affiliations:** Department of Microbiology, Nihon University School of Dentistry, Chiyoda-ku, Tokyo 102-8310, Japan

## Background

Latently infected cells harbor HIV-1 proviral DNA integrated in heterochromatins, allowing persistence of transcriptionally silent proviruses. Hypoacetylation of histone proteins by histone deacetylases (HDACs) is involved in maintaining the HIV-1 latency by repressing transcription from HIV-1 proviruses. Although it is known that a bacterial metabolite, butyric acid, is involved in reactivation of the ‘repressed’ chromatin, it is not known whether butyric acid-producing bacteria, such as periodontogenic *Porphyromonas gingivalis* and *Fusobacterium* in resident flora, are involved in activation of HIV gene expression. Here, we examined whether infection of these bacteria could facilitate progression of HIV infection by reactivating the latent HIV provirus.

## Methods

Human ACH-2 and OM10.1 cells, latently infected with HIV-1, were incubated with culture supernatant of various bacteria. HIV-1 proteins were detected by immunoblot and ELISA. Luciferase and chromatin immunoprecipitation assays were employed to analyze the transcription factors involved in HIV transcription. Butyric acid and other short chain fatty acids were measured by gas chromatography.

## Results

We found that the culture supernatant of periodontopathic bacteria, *P. gingivalis*, strongly induced HIV-1 replication via chromatin modification and these effects could be ascribed to butyric acid (1). Other bacteria producing butyric acid, such as *Clostridium*, *Fusobacterium*, and *Eubacterium* in the intestine, also accelerated replication of HIV-1. In addition, some anaerobic bacteria resident in vaginal mucous membrane such as *Peptoniphilus* and *Anaerococcus* could also induce HIV-1 replication. These bacteria produce high concentrations of butyric acid acting as a potent inhibitor of HDACs and induce histone acetylation, thus eventually leading to reactivation of HIV-1 in latently infected cells.

## Conclusions

Our observations indicate that butyric acid-producing bacteria are generally involved in AIDS progression by reactivating the latent HIV provirus. Thus, it is concluded that elimination of such bacterial infection would prevent the clinical progression of HIV infection.

## References

[1] Imai K, Ochiai K, Okamoto T (2009). Reactivation of latent HIV-1 infection by theperiodontopathic bacterium Porphyromonas gingivalis involves histone modification. J Immunol.

# Sub article: Extracellular HIV-1 TAT (ExTat) regulates bacterial superoxide dismutase (SOD) allowing upregulation of reactive oxygen species (ROS) in *Porphorymonas gingivalis* and *Fusobacterium nucleatum*

**Published:** 2012-05-23

**Authors:** Marni Cueno, Muneaki Tamura, Kenichi Imai, Megumi Hamadate, Kuniyasu Ochiai

**Affiliations:** Department of Microbiology, Nihon University School of Dentistry, Tokyo, Japan

Among AIDS patients, viral load has been detected in the saliva suggesting extracellular HIV-1 Tat (ExTat) also accumulates. ExTat is known to cause oxidative stress by regulating superoxide dismutase (SOD) activity, thereby allowing reactive oxygen species (ROS) to accumulate. The effects of ExTat is well studied in humans; however, its effects in the bacterial flora have never been elucidated. Here, we studied the effects of Tat on periodontal anaerobic bacteria. We compared the effects of commercial Tat (CommTat) and ExTat produced from plant cells on both *Porphorymonas gingivalis* ATCC 33277 and *Fusobacterium nucleatum* ATCC 25586. Bacterial isolates were grown in GAM broth supplemented with either CommTat or ExTat at a final concentration of 0.9 mg/mL. Both Tat-supplemented and control strains were grown under anaerobic conditions for 48 h at 37°C. Cells were lysed using 10% SDS solution. ROS and SOD levels were measured using commercially available assays. In both control and Tat-supplemented samples, we found no drastic changes in bacterial growth density; however, SOD levels were found to be downregulated, which we attribute to Tat action. Subsequently, ROS levels were found to be higher among Tat-supplemented samples as compared to control samples. Interestingly, we found ExTat induced higher ROS as compared to CommTat. We attribute this observation to glycosylation since, unlike CommTat where Tat is unglycosylated, Extat, which was produced from plant cells, is glycosylated and, thus, is more room-temperature stable. Our results would suggest that, at least among anaerobic bacteria, particularly *P. gingivalis* and *F. nucleatum*, Tat downregulates SOD activity, which subsequently allows ROS to accumulate. This would imply two points: (1) ROS increase ascribable to ExTat is not unique to mammalian cells and (2) the bacterial flora of HIV-infected patients may similarly be affected by ExTat.

# Sub article: Evaluation of survival and infectivity of bacteriophage isolates from environmental samples in function of temperature and pH

**Published:** 2012-05-23

**Authors:** Nima Bahador

**Affiliations:** Department of Microbiology, Islamic Azad University, Science and Research Branch, Fars, Iran

**Keywords:** Bacteriophage, survival, infectivity, temperature, pH

Twenty bacteriophages were isolated from Pavana river water and sewage samples (India). Out of all the isolates, two bacteriophages were characterized and identified (according to Ref. 1) as coliphage (Leviviridae) and staphylophage (Podoviridae). Then, effect of pH and temperature on survival and infectivity of the isolates were evaluated in order to achieve optimum pH and temperature for the isolates and use them in further study for eradication of biological pollutant in water. The buffers that have been used in this study were succinate, Tris and glycine. In addition, effect of different temperatures (20, 25, 30, 35, 40 and 45°C) on survival and infectivity of the phages have been evaluated. The results indicated that the optimum pH for both of the isolates was 7.0. The optimum temperature for survival and infectivity of the isolates were 37°C for coliphage (Ecp) and 27°C for the staphylophage (Sap). Overall, evaluation of optimum temperature and pH as significant character for eradication of microorganisms using bacteriophages are required.

## References

[1] Ackerman (1998).

# Sub article: Coinfection of mammalian and tick cells with pathogenic and nonpathogenic spotted fever group rickettsiae

**Published:** 2012-05-23

**Authors:** Tsuneo Uchiyama, Hiromi Fujita

**Affiliations:** 1 Department of Microbiology, Institute of Health Biosciences, The University of Tokushima Graduate School, Kuramoto, Tokushima, Japan; 2 Ohara Research Laboratory, Ohara General Hospital, Kamata, Fukushima, Japan

It is known that ticks sometimes possess multiple species of rickettsiae. However, it is still uncertain if coinfection of humans with these rickettsiae actually occurs. In this study, we intended to analyze the growth kinetics of pathogenic and nonpathogenic spotted fever group rickettsiae when they are coinfected in mammalian and tick cell lines.

*Rickettsia japonica* was used as a pathogenic species of spotted fever group rickettsiae. *R. montanensis*, isolated from ticks in the northern and eastern USA, and *Rickettsia* sp. LON, isolated from ticks in Japan and genetically closely related to *R. japonica*, were used as nonpathogenic rickettsiae. Vero and HeLa cells derived from mammals and DALBE3 and ISE6 cells derived from ticks were inoculated with nonpathogenic strains. On the day three of inoculation, the infected cells were further inoculated with pathogenic *R. japonica*. Inoculation in the reverse order was also performed. The yields of each rickettsia were measured by means of plaque assay and immunostaining with species-specific monoclonal antibodies. These cells were solubilized every three days for Western blotting to clarify the participation of autophagy in the growth of these rickettsiae. Transmission electron microscopy was also performed to observe their morphological changes.

The mammalian and tick cells were persistently infected with *R. montanensis* producing low levels of rickettsiae. The elevation of the yields of *R. montanensis* occurred after the superinfection with *R. japonica* on the *R. montanensis*-infected cells. On the other hand, LON grew well in tick cells although it caused persistent infection in mammalian cells. After the superinfection with *R. japonica* on the LON-infected mammalian cells, the elevation of the yields of LON also occurred. The cause of the difference in the growth kinetics of *R. montanensis* and LON in tick cells is yet to be elucidated. Western blotting and the electron microscopy presented the evidences of autophagy occurred in the *R. montanensis*-infected cells. After superinfection with *R. japonica*, autophagy was rather restricted. It is suggested that the growth restriction of the nonpathogenic species *R. montanensis*is at least partly due to the autophagy that occurs in the infected cells and that the pathogenic species *R. japonica* may secretes an autophagy-restriction factor(s). The factors are yet to be identified.

# Sub article: *Leptotrichia* genus bacteria biotopes in the human body

**Published:** 2012-05-23

**Authors:** Natalia Viktorovna Strelnikova, Alexandra Anatolievna Antonova, Elena Borisovna Polovova

**Affiliations:** Microbiology, Virology and Immunology Department, Far Eastern State Medical University, Khabarovsk, Russia

*Leptotrichia* are nonspore-forming gram-negative anaerobic rod and are normal commensals of the human oropharynx, gastrointestinal tract and female genital tract. *Leptotrichia buccalis* is the type species of *Leptotrichia* genus and since 2005 belongs to *Fusobacteriaceae* family.

*L. buccalis* is the species most frequently isolated from clinical samples. *Leptotrichia* species are considered an important component in mixed anaerobic infections. Infection caused by *L. buccalis* is rare.

Nevertheless, according to the literature over the past 20 years, the role of *Leptotrichia* genus bacteria inhuman pathology is increasing (1–15).

At present, *Leptotrichia* genus bacteria can be regarded as emergent pathogens in the infectious pathology of man (16, 17). The organism has been isolated mostly from opportunistic infections. The most frequently isolated bacteria from blood samples in cases of patients with lesions in the oral mucous membrane, in particular from patients with neutropenia and cancer. *Leptotrichia* has also been recovered from immunocompetent patients with severe pneumonia, hepatic abscess, endocarditis, bacterial vaginosis and other. Although their role in infections is poorly understood, they have been suggested as emerging pathogens.

In our study of *Leptotrichia* infections in adult and children, a total of 43 strains of *Leptotrichia* species were recovered from 229 (18, 8%) specimens. In one case, *Leptotrichia* has been isolated from blood culture of immunocompetent 19-year-old girl with rectal fistula. In one case, *Leptotrichia* has been isolated from plural hepatic abscesses culture of 53-year-old woman. In a series of 24 patients with chronic tonsillitis, *Leptotrichia* and *Staphylocococcus* were isolated in 13 cases and *Leptotrichia* and *Candida* in 6 cases; *L. buccalis* alone was isolated in only 5 cases.

As the findings show, *Leptotrichia* species were recovered from blood culture of female with bronchial asthma and fever. In addition, isolated *Leptotrichia* from urine samples in cases of patients with chroniccystitis, pyelonephritis and urolithiasis and in particular from patients with congenital renal pathology.

*Leptotrichia* are normal commensals of the human oral microbiota and, in Khabarovsk territory, are found in 95% of the population. Under certain conditions, *Leptotrichia* can cause inflammatory diseases of the oral mucosa, e.g., stomatitis, gingivitis, cheilitis and glossitis. The etiology of about 34% of the cases presented *Leptotrichia* and association of microbes: *Leptotrichia* with *Staphylocococcus*, *Leptotrichia* with *Candida*, *Leptotrichia* with *Enterococcus*.

These data require a deeper study of condition immunity, as well as environmental factors affecting the state of oral microbiota in the population of the region.

## References

[1] Moore WE, Holdeman LV (1982).

[2] Duperval R (1984).

[3] Patel JB (1999).

[4] Saito T (2003).

[5] Fredricks DN (2005).

[6] Ulstrup AK (2006).

[7] Goto M (2007).

[8] Boennelycke M (2007).

[9] Thilesen CM (2007).

[10] Tamrakar R, Yamada T (2007).

[11] Caram LB (2008).

[12] Hot A (2008).

[13] Zhang Y (2008).

[14] Adriaens LM (2009).

[15] Kawanami T (2009).

[16] Blairon L (2006).

[17] Eribe ERK, Olsen I (2008).

# Sub article: Nasopharyngeal microbial biocenosis in children in the Amur River area of Russia

**Published:** 2012-05-23

**Authors:** Galina Nikolaevna Kholodok, Vladimir Kirillovich Kozlov

**Affiliations:** Research Institute of Mother and Child Health Care, Khabarovsk Branch of the Far-Eastern Research Center of Respiratory Physiology and Pathology under the Siberian Branch of the Russian Academy of Medical Sciences

Microbial biocenosis is important for maintenance of homeostatic balance in the human body (1). It is characterized by stability, which is due to Quorum sensing bacteria (2, 3), associated in a microbial-tissue complex, a biofilm (4). Respiratory tract microbiota accounts for the macroorganism's colonization resistance, while its disbalance leads to a risk of pneumonia. Our own research has shown that the extreme biogeoclimatic environment in the Russian Far East contributes to an increase in respiratory diseases in children (5, 6), which, among other things, is due to the nasopharyngeal circulation of opportunistic bacteria in the regional children population.

The objective of the research consisted in the study of respiratory microbial biocenosis and its assessment thereof as a risk factor of community-acquired pneumonia in children.

Nasopharyngeal microbial biocenosis in 1,175 children was studied; 1,640 strains of indigenous and transitory opportunistic bacteria of 8 families, 14 genera and 32 species were isolated. To assess species diversity in a nasopharyngeal biotope, Simpson index, Margalef species richness index calculated as a ratio of the number of species to the total number of individuals in the biotope, Shannon (entropy) index providing for the number of species and the extent of their activity in the community and attaching greater importance to rare species were used. Pielou index admitting of diversity comparison among the samples with different number of species and assessment of the degree of community polydominance, Berger–Parker index representing the number of the most abundant species in the community were also used.

Analysis of the data obtained shows that Shannon index indicates to the stable composition of *pharyngeal* microbiota due to the presence of gram-positive bacteria, with their diversity (assessed by Simpson index) being less than that of gram-negative bacteria. Shannon index for gram-positive flora in the pharynx made 1.16, for gram-negative flora in the pharynx, 0.56 (i.e., 2 times as less) with Simpson's index equal to 0.37 and 0.71 and Margalef index equal to 1.69 and 1.28, respectively. Berger–Parker index in the pharynx showed prevalence of genus *Neiseriae* (0.69) among gram-negative bacteria, genus *Streptococcus* (0.468) among gram-positive bacteria, and genus *Acinetobacter* (0.8) among nonfermentative bacteria. For nasal microbiota, gram-negative flora was of greater significance. Shannon index for gram-positive bacteria was found to be less than for gram-negative bacteria and made 0.97 and 1.02, with Pielou evenness indices being 0.49 and 0.73, respectively. Species diversity in nasal microbial biocenosis was greater for gram-positive bacteria. Simpson's index made 0.52 and 0.45 and Margalef's index, 2.6 and 1.89 for gram-positive and gram-negative bacteria, respectively. Berger–Parker indices in a nasal biotope were high for *Staphylococci* (0.7) and *Neiseriae* (0.61). Thus, a dysbiocenotic state of a nasopharyngeal microbiota was identified, which was caused by increased enterobacterial and staphylococcal vegetation. Nasopharyngeal carriage of *Streptococcus pneumoniae* and *Haemophilus influenzae* in organized children groups made 36.3% and 49%, and, in case of PCR detection of DNA, made as much as 92.5% and 70.2%, respectively. *Staphylococcus aureus* carriage was identified in 23.7% cases (up to 50% in some categories of children). Enterobacteria in a nasopharyngeal microbiota of the population are detected in 2.4±0.37% cases, which is significantly more rare than in pneumonic children—in 13.9±1.2% cases (*p*<0.001). The level of nasopharyngeal carriage of *S. pneumonia* and *H. Influenzae*, estimated by bacteriological and PCR methods, was found to be 36% and 86.7% of cases and 92.5% and 70.2% of cases, respectively. *Moraxella catarrhallis* DNA was detected in 73% of cases. The nature of discovered changes in microbial biocenosis in children of the Amur River area evidences of low colonization resistance of respiratory tract microbiota and a high risk of bacterial inflammation in the respiratory system.

## References

[1] Kozlov VK, Rakitskaya EV, Krasnova MA, Uchakina RV (2006). Actual issues of adolescent medicine/Collect. abstr. Scientific-prakt. Konf. with international participation “Actual problems of the health of women and children at this stage,” October 31-November 1, 2006. Khabarovsk.

[2] Kozlov VK, Evseeva GP (2010). Medical and demographic characteristics and health status of children in the Far Eastern Federal District / Mater. First Congress of pediatricians of the Far East,
20–21 May 2010. Khabarovsk.

[3] Tkachenko EI, Uspensky YP (2006). Nutrition, microbiocenosis and the human intellect/St.

[4] Donlan RM (2002). Biofilms: surviral mechanisms of clinically relevant microorganisms. Clin Microbiol Rev.

[5] Kievit TR, Iglevski BH (2000). Bacterial quorum sensing in pathogenic relationgships. Infect Lmmun.

[6] Miller MB, Bassler BL (2001). Quorum sensing in bacteria. Annu Rev Microbiol.

# Sub article: Implication of the role of stringent response in the expression of adenylate cyclase toxin in *Bordetella pertussis*

**Published:** 2012-05-23

**Authors:** Tomoko Hanawa, Kentaro Sugisaki, Hideo Yonezawa, Takako Osaki, Satoshi Kurata, Cynthia Zaman, Shigeru Kamiya

**Affiliations:** Department of Infectious Diseases, School of Medicine, Kyorin University, Shinkawa Mitaka, Tokyo, Japan

*Bordetella pertussis* is an etiological agent of whooping cough and produces a lot of virulence factors such as adhesins and toxins. The transcriptions of major virulence factors such as adenylate cyclase toxin (ACT), pertussis toxin and filamentous hemagglutinin are regulated by BvgSA two-component system. It is modulated by concentration of MgSO_4_, nicotinic acid or unknown factor(s) in the host environment.

ACT is an essential virulence factor for *B. pertussis* and other *Bordetella* species. This toxin is a bifunctional protein exhibiting both adenylate cyclase and hemolysin enzymatic domains and able to invade target cells and to impair intracellular functions. ACT is encoded by *cyaA*, and in the same operon, there are three genes, *cyaB, cyaD* and *cyaE*, encoding the type I secretion machinery members. After synthesis of a 177-kDa precursor and palmitoylation by CyaC, ACT is secreted as a 216-kDa active form by type I secretion system.

Stringent response is a most important stress response to the nutritional starvation. When amino acids or carbon sources are depleted, ppGpp molecules are accumulated in the cell and regulate various genes transcriptions positively or negatively. In consequence, growth rate is repressed to avoid death by overgrowth under the limited nutrients.

In the present study, we found the increase of both cellular and secreted ACT by deletion of ppGpp synthetases in *B. pertussis*. Our findings suggest the regulation of expression of ACT by stringent response in *B. pertussis*.

# Sub article: Examination of growth conditions and nitrogen fixation capability of legumes selected from various sources in Northern Greece

**Published:** 2012-05-23

**Authors:** Stavros Kazakos, Stavros Plessas, Athanasios Alexopoulos, Eugenia Bezirtzoglou

**Affiliations:** 1 Democritus University of Thrace, Faculty of Agriculture Development, Laboratory of Microbiology, Biotechnology and Hygiene, Orestiada, Greece; 2 Democritus University of Thrace, Faculty of Agriculture Development, Laboratory of Food Processing, Orestiada, Greece

Legumes are a very significant research field the last years due to the great importance of nitrogen fixation. In the frame of the present research work, various legumes were collected: *Cicer arietinium, Glycine max, Lathyrus sativus, Lathyrus clymenym, Lens culinaris, Lupinus albus, Pisum sativum* and *Vicia faba*. Afterward, nitrogen-fixing bacteria were isolated from legumes, and the possibility of nitrogen fixation was studied *in vitro* using the appropriate synthetic liquid (Yeast extract mannitol), as well as the conditions of growth in various acidities conditions. The results showed that the symbiotic relation between nitrogen-fixing bacteria and nodules is of great importance for the function of nitrogen fixation and that this function does not seem to work *in vitro*. Regarding the acidity study, it was clear that nitrogen-fixing bacteria were cultured successfully and that they have the same demands to those that have in the case of soil, with some small differences. In general, it was revealed that they do not grow in acidic conditions and they prefer pH values near to 7. Nevertheless, in the case of *L. culinaris* and *G. Max*, the highest biomass production was determined at pH value of 5. The explanation is due to the kind of bacteria isolated in the laboratory, since there are cases of bacteria such as *Rhizobium loti* and *R. tropici* that are resistant in low levels of pH. In general and according to international studies, it has been supported that different kinds of the same genus may show different in different levels of acidities.

# Sub article: Biochemical studies on the virulence factors of fungi associated with she-camel milk

**Published:** 2012-05-23

**Authors:** Abd El-Aziz Mosaad, Ahmed El-Kirdasy, Mostafa Al-Sherif, Said Fathalla, Abd El-Rahman M El-Bagory, Gamal Abd El. Gaber

**Affiliations:** 1 Department of Bacteriology, Mycology and Immunology, Faculty of Veterinary Medicine, Minufiya University (Sadat Branch), Egypt; 2 Department of Biochemistry, and Chemistry of Nutrition, Faculty of Veterinary Medicine, Minufiya University (Sadat Branch), Egypt; 3 Department of Biology, Faculty of Art and Science, Al-Mergeb University, El-khoms, Libya; 4 Department of Physiol., Faculty of Vetinary Medicine, Minufiya University (Sadat Branch), Egypt; 5 Department of Food Hygiene & Control, Faculty of Vetinary Medicine, Minufiya University (Sadat Branch), Egypt; 6 Department of Bacteriology, Mycology and Immunology, Faculty of Vetinary Medicine, Mansoura University, Egypt

**Keywords:** Camel milk, yeast

Milk is an ideal habitat for the growth and multiplication of microorganisms due to its nutritional constitution which contains protein, carbohydrate, mineral and vitamins. All these components support the growth of many forms of bacteria, fungi and yeasts. Raw milk aseptically drawn from a healthy animal usually contains a few bacteria. This work aimed to study the virulent factors of isolated yeasts in raw camel milk. A total of 40 samples were collected from camel milk from different localities in Egypt and were subjected for subclinical mastitis ‘California mastitis test’. Results revealed that four samples (10%) have subclinical mastitis and the rest of samples were negative to the test. From the results of fungal examination and isolation on specific media, we found that six samples were single infection (15%) and two were mixed infection (5%). Identification of these isolates were *Aspergillus niger* 1 (2.5%) *Aspergillus fumigatus* 1 (2.5%), *Rhizopas* 1 (2.5%), *Mucor* 1 (2.5%), *Penicillium* 2 (5%) and *Candida albicans* 4 isolates (10%). We found that all *C. albicans* have ability to produce germ tube by incubation on rabbit serum, Chlamydospore, and phospholipase enzyme by using PCR to detect this enzyme as a rapid and sensitive method, so we found that all isolates have ability to produce that enzyme and we can use PCR also as a direct method for Identification with high efficacy. From the results, it can be concluded that: low incidence of fungal agents associated with she-camel's milk and the main common moulds and yeasts associated with mastitis were zygomycetes ‘*Rhizopus* 1 (2.5%), *Mucor* 1(2.5%)’, *A. fumigatus* 1 (2.5)%, *A. niger* 1 (2.5%), *Penicillium* 2 (5%)’ and *C. albicans* 4 (10%). Many virulent factors of *C. albicans* can be detected and produced such as germ tube, chlamydospores. Also, proteolytic and phospholipase activities can be detected by using PCR.

# Sub article: *In vivo* antibacterial activity of Phx-3 against *Helicobacter pylori*

**Published:** 2012-05-23

**Authors:** Fuhito Hojo, Takako Osaki, Tomoko Hanawa, Akio Tomoda, Shigeru Kamiya

**Affiliations:** 1 Institute of Laboratory Animals, Graduate School of Medicine, Kyorin University, Tokyo, Japan; 2 Department of Infectious Diseases, Division of Medical Microbiology, Kyorin University, School of Medicine, Tokyo, Japan; 3 Department of Biochemistry and Intractable Immune System Disease Research Center, Tokyo Medical University, Tokyo, Japan

Phx-3 (2-amino-phenoxazine-3-one), one of the phenoxazine derivatives, is reported to have inhibitory effect on several pathogens. We have demonstrated that Phx-3 has antibacterial activity against *Helicobacter pylori in vitro*, but the effect of Phx-3 on *H. pylori in vivo* have not been assessed. Then, we examined the *in vivo* antibacterial activity of Phx-3 by using the mice infected with *H. pylori* Sydney Strain 1 (SS1). In the present study, it has been shown that colony-forming unit of *H. pylori* isolated from stomach significantly reduced by Phx-3. In addition, histological analysis has shown that plasma cells and Russel bodies were observed in gastric submucosa of mice treated with Phx-3, indicating induction of humoral immunity.

# Sub article: Detection of *Campylobacter* is possible using their physical characters

**Published:** 2012-05-23

**Authors:** Majid Baserisalehi

**Affiliations:** Department of Microbiology, Islamic Azad University-Kazeroun branch, Kazeroun, Iran

The major aim of this study was introduced the novel device (Kapadnis–Baseri device) and method (prêt-Kapadnis Baseri) for isolation of *Campylobacter* spp without using selective media incorporated antibiotics. In the present study, detection of these bacteria was carried out using their physical characters (viz., motility and its activity at law temperature) instead of their antibiotic resistant character. To determine ability of these methods for isolation of *Campylobacter*, their isolation rate was evaluated in comparison with the conventional methods.

In total, 238 environmental samples (animal feces, viz., cow and sheep) were collected and 68 *Campylobacter* strains were isolated from them. Of all isolates, 39 and 19 strains were isolated by the new methods and conventional methods, respectively. Therefore, the rate of *Campylobacter* isolation using the new methods was high in comparison with conventional methods. Overall, the KB device and the prêt KB method were designed and developed on the basis of unique characters of *Campylobacter*, viz., motility and its activity at low temperature (6°C). It means that the KB device and the prêt KB method minimize population of competing bacteria and detect *Campylobacter* from the environmental samples without using antibiotics. Hence, based on foregoing evidence, the new device and the method will be useful for isolation and enumeration of *Campylobacter* from environmental samples. Besides, these methods would also detect antibiotic sensitive campylobacters, which are not detected by conventional methods.

# Sub article: Bacillus probiotics: biological and clinical effects

**Published:** 2012-05-23

**Authors:** Larisa A. Safronova

**Affiliations:** Zabolotny Institute of Microbiology and Virology, Kiev, Ukraine

Production of effective and safe for human and environment biological preparations for medicine and agriculture on the basis of microorganisms is quickly growing area of biotechnological market. Use of such preparations promotes strengthening of human health and does not lead to negative consequences for an organism and an environment.

The bacteria of the genus *Bacillus* are widely occurred in the environment, including those objects, with which human and the animals are in constant contact—in air, water, ground, feeds and food products. Many strains of aerobic sporeforming of bacteria are characterized by high biological activity. They synthesize such biologically active substances, as enzymes, amino acids, vitamins, antibiotics, lectinis, polysaccharides and other compounds. Among bacteria of the genus *Bacillus* are quite often found strains with antagonistic effect against pathogenic and conditionally pathogenic microorganisms isolated from various biotopes.

Probiotics developed on the basis of aerobic sporeforming of bacteria of the genus Bacillus is used for a prophylaxis and treatment of dysbacteriosis, number of infectious diseases, as immunomodulators. But, certainly, more research is needed on the mechanisms of probiotic action of aerobic sporeforming bacteria.

In the institute of Microbiology and Virology of NAS of Ukraine, a number of preparations containing live cultures of bacilli for medicine and agriculture are developed. High efficacy and safety of probiotic preparations have been shown. Probiotics biosporin and subalin are successfully used in medical practice.

# Sub article: Monoclonal antibodies against the accumulation-associated protein influence EPS biosynthesis and enhance bacterial accumulation of *Staphylococcus epidermidis*

**Published:** 2012-05-23

**Authors:** Yang Wu, Jian Hu, Tao Xu, Huayong Liu, Youcong Wu, Di Qu

**Affiliations:** Key Laboratory of Medical Molecular Virology of Ministries of Education and Health, Institute of Medical Microbiology and Institutes of Biomedical Sciences, Fudan University, Shanghai, China

**Keywords:** Staphylococcus epidermidis, biofilm formation, extracellular polymeric substance, accumulation-associated protein, monoclonal antibody, epitope

*Staphylococcus epidermidis* is a normal inhabitant of human skin and mucous membranes that rarely causes pyogenic infections in healthy individuals. However, during the past two decades, *S. epidermidis* has emerged as one of the major pathogens in nosocomial infections. The primary pathogenicity trait of *S. epidermidis* is associated with its ability to form biofilms on surfaces of medical devices, limiting severely the efficacy of many conventional antibiotics, and biofilms may also protect the bacteria against attacks from the host defense system. Because, to date, there is no effective antibiotic to eradicate biofilm infection, the development of biofilm-preventing vaccines is necessary.

Biofilm formation by *S. epidermidis* requires accumulation-associated protein (Aap), which is considered one of the most important protein-based adhesins. Aap contains sequence repeats known as G5 domains. The zinc-dependent dimerization of G5 domains could be responsible for the function of Aap to mediate the intercellular adhesion of *S. epidermidis*. Antibodies against Aap have been reported to inhibit biofilm accumulation by *S. epidermidis*, indicating that Aap could serve as a vaccine candidate to prevent *S. epidermidis* biofilm infection. However, the full-length Aap is not considered to be a safe vaccine for systemic immunization because such bacterial antigens contain many antigenic determinants and may induce hypersensitivity reactions. A peptide that induces antibiofilm humoral immunity would be an optimal vaccine. The Aap *C*-terminal single B-repeat construct followed by the 79-aa half repeat (AapBrpt1.5) is the basic functional unit of Aap, which is necessary to mediate bacterial accumulation, suggesting that it harbors the epitopes that would guide the development of biofilm-preventing epitope-based peptide vaccines.

To locate the epitopes in Aap that induce antibiofilm humoral immunity, monoclonal antibodies (MAbs; MAb_18B6_, MAb_25C11_ and MAb_20B9_) against AapBrpt1.5 were prepared in the present study. All of the MAbs we generated bound to Aap antigens at extremely high affinities. However, only MAb_18B6_ was found to possess the weak but broad-spectrum activity necessary to inhibit biofilm formation by *S. epidermidis*, whereas MAb_25C11_ and MAb_20B9_ enhanced the biofilm formation by some strains of *S. epidermidis*, including RP62A. All of the MAbs strengthened the cell aggregation of planktonic *S. epidermidis*. The epitope of each MAb was identified by using protein truncation and immunoprecipitation. Epitope mapping of the MAbs revealed that the biofilm-inhibiting MAb_18B6_ recognized an identical area within all Aap-Brpt constructs from *S. epidermidis* RP62A, which was not shared by the other two MAbs. To explore the reasons for the biofilm-enhancing activities of the MAbs, EPS biosynthesis was studied by detecting Aap expression, extracellular DNA release and PIA synthesis in *S. epidermidis* RP62A co-cultured with the MAbs. All MAbs were found to affect EPS biosynthesis in *S. epidermidis* and further enhance the bacterial accumulation. The different activities of the MAbs on biofilm formation could be related to the resultant effect of inhibition of Aap dimerization (inhibiting biofilm formation) and alteration of EPS biosynthesis (enhancing biofilm formation).

In addition to our novel findings on the altered EPS biosynthesis in *S. epidermidis* mediated by mouse MAbs against Aap, the epitope mapping of biofilm-affecting MAbs will, for the first time, contribute to a better understanding of staphylococcal biofilm formation and help develop epitope-peptide vaccines against staphylococcal infections.

# Sub article: Eubiotics in medicine

**Published:** 2012-05-23

**Authors:** Nina N. Gavrilova, Irina A. Ratnikova

**Affiliations:** The Institute of Microbiology and Virology, Almaty, Kazakhstan

## Plantafermin

The increased incidence of dysbacteriosis observed in the last decade among more than 90% of population is associated with environmental degradation, uncontrolled use of antimicrobial drugs, stress, poor food quality and other factors due to which it can be considered a social phenomenon. Intestinal dysbiosis weakens body defenses, increases susceptibility to infectious diseases and causes allergic reactions (exudative diathesis, food allergy and seborrhea). Intestinal dysbiosis leads to protracted, recurrent course of diseases, development of complications, disturbance of digestion and absorption of food.

Lactic acid and bifidobacteria preparations are currently proposed for struggling with dysbacteriosis: Bifidumbacterine, Lactobacterine, Linex, etc. However, they are not much efficient possibly due to low range of probiotic bacteria antimicrobial activity.

The Institute of Microbiology and Virology of the Ministry of Education and Science of the Republic of Kazakhstan formulated a broad-spectrum therapeutic drug Plantafermin based on lactic acid bacteria and bifidobacteria. The eubiotic drug is effective against dysbacteriosis of various etiology in children from infancy and adults, inflammatory and infectious diseases; in treatment of gastric and intestinal dyspepsia, defecation disorders, inflammatory and infectious diseases of gastrointestinal tract, liver and bile passage diseases, inflammatory and functional disorders of pancreatic gland; in treatment of inflammatory and infectious urogenital diseases; in treatment of candidiasis of different localization, its prevention in the period of antibiotic treatment, hormonal, cytostatic and immunosuppressive drug administration; for prevention of dysbacteriosis in stress, increased mental and physical tension under unfavorable environmental conditions.

## Fermented Beet Juice

Beet juices and drinks are useful in dietary nutrition due to the content of proteins, carbohydrates, fiber, free organic acids, potassium, sodium, vitamins B1, B2, PP, provitamin A and vitamin C in optimum ratios. The presence of polyphenols predetermines its use in irradiation injuries, cancerous, cardiovascular diseases and atherosclerosis.

We have developed a technology for production of beet juice fermented by specially selected strains of lactic acid bacteria. In the fermented juice: the content of coloring agent bethanidine is stabilized, and the quantity of essential amino acids and vitamins is increased—lysine by 25%, threonine by 60%, valine by 14%, isoleucine by 33%, leucine by 50% phenylalanine by 15%, vitamin B1 by 60%, vitamin B2 by 50%, vitamin C by 27%, water-soluble pectins by 1% with simultaneous reducing of nitrate content. Clinical trials carried out at the Nutrition Research Center of Kazakhstan showed that fortified fermented juice has positive therapeutic efficacy in treatment of chronic diseases: gastritis, enterocolitis, hepatitis, cholecystitis, and can be used as a curative drink.

# Sub article: Early and quantitative assay to detect HHV-6 viremia and evaluation of cellular response specific against HHV-6 after hematopoietic stem cell transplantation

**Published:** 2012-05-23

**Authors:** Shunichi Kato, Hiromasa Yabe, Chie Nakashioya, Fumiko Tsuchida, Tatsuya Sugimoto, Kimiyasu Shiraki, Masayuki Saijo

**Affiliations:** 1 Department of Cell Transplantation and Regenerative Medicine, Tokai University Hospital, Isehara, Japan; 2 Department of Virology, University of Toyama, Toyama, Japan; 3 Department of Virology 1, National Institute of Infectious Diseases, Tokyo, Japan

## Purpose

HHV-6 infection is one of the major morbidity and mortality after hematopoietic stem cell transplantation (HSCT), and early diagnosis of HHV-6 viremia and evaluation of specific cellular immunity are needed.

## Methods and Patients

1. HHV-6 viremia was assayed for 933 blood samples of 131 allogeneic HSCT recipients by a real-time PCR test, which was designed to specifically detect HHV-6.

2. HHV-6-specific cellular immunity was evaluated using peripheral blood mononuclear cells of 114 allogeneic HSCT patients by an LPR assay. Normal healthy volunteers served as positive controls and 11 cord blood donors as negative controls.

## Results

1. HHV-6 was frequently detected in the blood samples in the first 2 to 6 weeks after HSCT.

2. HHV-6-specific LPR became positive 4 to 8 weeks after HSCT following HHV-6 viremia, and HHV-6 viremia usually disappeared following positive LPR, but low number of HHV-6 copies could be detected in a small portion of patients for months after HSCT.

## Conclusion

HHV-6 infection could be specifically and quantitatively in HSCT patients.

This work was supported in part by the Health and Labour Sciences Research Grant(s) for research on measures for emerging and reemerging infections.
